# The Smartphone—Not So ‘Smart’ for Teenagers’ Health

**DOI:** 10.3390/children13101402

**Published:** 2026-10-14

**Authors:** Reka Borka Balas, Lorena Elena Melit, Dora Sarolta Balint, Dana Ghiga, Anna Borka Balas, Hajnal Finta, Cristina Oana Marginean

**Affiliations:** 1 Department of Pediatrics I, George Emil Palade University of Medicine, Pharmacy, Science and Technology of Targu Mures, 38 Gheorghe Marinescu Street, 540139 Targu Mures, Romania; reka.borka-balas@umfst.ro (R.B.B.); oana.marginean@umfst.ro (C.O.M.); 2 Pediatric Clinic I, County Emergency Hospital Targu-Mures, 50 Gheorghe Marinescu, 540136 Targu Mures, Romania; dora_balint@yahoo.com; 3 Department of Pediatrics II, George Emil Palade University of Medicine, Pharmacy, Science and Technology of Targu Mures, 38 Gheorghe Marinescu Street, 540139 Targu Mures, Romania; 4 Pediatric Clinic II, County Hospital Targu-Mures, 6 Bernady Gyorgy Pta, 540072 Targu Mures, Romania; 5 Department of Research Methodology, George Emil Palade University of Medicine, Pharmacy, Science and Technology of Targu Mures, 38 Gheorghe Marinescu Street, 540139 Targu Mures, Romania; dana.ghiga@umfst.ro; 6 Faculty of Medicine, George Emil Palade University of Medicine, Pharmacy, Science and Technology of Targu Mures, 38 Gheorghe Marinescu Street, 540139 Targu Mures, Romania; borka-balas.anna.23@stud.umfst.ro; 7 Department of Public Health and Health Management, George Emil Palade University of Medicine, Pharmacy, Science and Technology of Targu Mures, 38 Gheorghe Marinescu Street, 540139 Targu Mures, Romania; hajnal.finta@umfst.ro

**Keywords:** smartphone, excessive smartphone use, problematic smartphone use, children, adolescents, sleep disturbances, depression, anxiety

## Abstract

**Highlights:**

**What are the main findings?**

- Longer daily smartphone use was associated with less favorable sleep-related outcomes.
- The patterns of association differed between age groups. Among younger children, smartphone use before bedtime was associated with insufficient and non-restorative sleep, whereas among adolescents, longer daily smartphone use was associated with insufficient sleep and disturbing dreams.
- Smartphone use patterns differed by age and sex, with adolescent girls reporting significantly longer daily use than boys.

**What are the implications of the main findings?**

- These findings highlight the importance of considering both the duration and timing of smartphone use when assessing sleep-related and physical complaints in pediatric populations.
- Age-appropriate guidance on smartphone use, particularly around bedtime, may be relevant for promoting healthy digital habits and sleep hygiene.
- Longitudinal studies using validated assessment instruments and objective measures of smartphone use are needed to clarify the direction and clinical significance of these associations.

**Abstract:**

Background: Smartphones have become indispensable tools for communication, entertainment, and information access among children and adolescents. Excessive smartphone use may interfere with daily activities and contribute to problematic smartphone use. This study aimed to investigate age-related differences in smartphone use patterns and their impact on health among children and adolescents. Methods: A cross-sectional, online questionnaire-based study was conducted between March and May 2021 among 415 smartphone users aged 7–18 years. Participants were divided into two age groups (group 1: 7–12 and group 2: 13–18 years). Sociodemographic characteristics, smartphone use patterns, lifestyle factors, and health-related outcomes were assessed. Statistical analyses were performed in R version 4.3.3. Results: In group 1, daily smartphone use was mostly 1–3 h (40.35%), whereas in adolescents it was 3–5 h (40.85%). Female adolescents spent significantly more time using smartphones than males (*p* < 0.0001), whereas boys had higher odds of using their smartphone for gaming than girls (aOR = 3.55, 95% CI 1.83–7.09). Approximately two-thirds of adolescents in both age groups reported excessive daily smartphone use and perceived themselves as “addicted”. Ocular complaints were among the most frequently reported somatic symptoms, particularly among adolescents. Prolonged smartphone use was significantly associated with disturbing dreams OR = 1.89, 95% CI (1.30–2.81) and insufficient sleep OR = 1.60, 95% CI (1.11–2.34) in adolescents; smartphone use before bedtime was significantly associated with daytime sleepiness in both groups (group 1 *p* = 0.041, group 2 *p* = 0.040). Anxiety-related symptoms were reported by nearly all participants, while emotional symptoms/experiences suggesting eventual depression appeared more prevalent among younger children. Conclusions: Excessive smartphone use is highly prevalent among children and adolescents and is associated with sleep disturbances, somatic symptoms, and psychological complaints, highlighting the need for targeted preventive interventions.

## 1. Introduction

The smartphone has become a mandatory object in the life of people regardless of their age. Thus, these devices represent not only an essential mean of communication, but also a major source of entertainment and information. Adolescents and young children spend a lot of time with their smartphones browsing social media, especially Facebook, Instagram, and Twitter. [1] It was recently underlined that up to 98% of young people aged between 15 and 24 years living in developed countries and 66% of those from developing countries have reported to use the internet every day [2]. Overall, 6.4 billion people worldwide have reported to use smartphones in 2021 and this number is expected to raise to 7.5 billion in 2026 [3]. It is true that smartphones enable internet access in any environment and have made our lives easier in terms of communication, socialization, entertainment, and access to information, and even improve time management when used judiciously [4,5]. The dark side of smartphone and internet use is defined by excessive and problematic smartphone use and smartphone addiction. There is considerable heterogeneity in the terminology and scales assessing problematic smartphone use and smartphone addiction [6,7]. Excessive smartphone use refers to a high frequency or duration of smartphone use that exceeds what is considered appropriate or necessary for an individual’s age or daily activities and is primarily a quantitative concept. Smartphone use by itself does not imply addiction or clinically significant impairment. Problematic smartphone use refers to a pattern of smartphone use characterized by impaired control or difficulty regulating use, which may interfere with everyday functioning and may be associated with adverse social, educational, physical, or psychological consequences (depression, anxiety, chronic stress, and/or low self-esteem) [8]. No consensual definition of smartphone addiction (SA) exists, which is why it is considered to be a behavioral addiction based on the classic addiction symptomology: a compulsive state in which an individual’s physiological, psychological, and/or social functions are impaired due to the uncontrolled use of smartphones [7,9].

Internet addiction is a commonly used term in the current era that is used to define a person’s inability to control internet use, resulting in an impairment of the psychological state and major health problems in daily life [10,11]. Internet addiction is in fact associated with a wide-spectrum of negative life quality effects involving health impairment, such as stress, anxiety disorders, social anxiety, depression, self-identity confusion, attention deficit/hyperactivity disorder, low-self-esteem, and even suicide attempt [12,13,14,15,16]. Likewise, problematic smartphone use seems to be associated with several negative health outcomes, especially in teenagers, such as lack of physical energy, immunity deficits, and physiological disfunctions, which are mostly related to the sedentary lifestyle associated with excessive smartphone use [17,18]. Therefore, it is not surprising that smartphone use is not necessarily only related to positive effects, especially in young people, since problematic smartphone use was defined by similarity as excessive use of these devices that disrupts the users’ daily life, involving symptoms like checking the phone frequently, expressing an urgent need to have the phone nearby, insomnia, and poor sleep quality [17,19,20,21]. Several components of smartphone addiction were recently described, such as tolerance, withdrawal, compulsive behavior, and functional impairment [22,23].

Adolescence might be defined as a vulnerable period of a person’s life when addiction is a predominant trait [24]. Across time, several defining elements have been proposed in order to define addictive behavior, such as control loss, tolerance, the establishment of a dependent relationship, alterations in mood, abstinence, personal harm, conflicts with peers, difficulty in avoiding sadness, the uncontrolled need for increasing the amount of time and dedication for a particular activity, major interference with daily life activities and quality, and last but not least, the tendency to relapse [25,26]. Moreover, the constant changes that occur in teenagers’ lives do not occur at the same age for all teenagers. Moreover, these changes, especially the psychological ones, should be closely monitored by parents, peers, and even health care providers in order to preempt their perpetuation and negative effects on youth quality life. In addition, a recent study proved that teenagers are not the only ones who are affected by excessive smartphone use, but in fact these devices might also impact the sleep quality of children aged between 1 and 14 years [27,28]. Adolescents might be called digital natives since they prefer to express their feelings in an online space, aiming to keep up with fashion, as well as searching for support and emotional relationships [29,30]. Unsurprisingly, teenagers need a private online space where they can access all the information they need or where they can use the desired apps [29]. Teenagers tend to gain independence from their parents, especially regarding the psychological aspects, while at the same time expressing the desire to increase the level of attachment with their peers, a fact that leads to a positive impact on psychological health, including self-esteem and resilience [31,32]. Smartphones enhance the link between adolescents and peer groups, resulting unfortunately in excessive or problematic smartphone use. Moreover, the proper use of these “smart” devices is in fact associated with the positive development of self-identity, which also increases sociality and creativity [33,34]. Nevertheless, teenagers with problematic smartphone use or smartphone addiction tend to isolate themselves and socialize only online, which is associated with an increased risk for cyber-delinquency [35,36]. Thus, adolescents that solely express a virtual socialization pattern tend to avoid “real-life” stress due to their low self-esteem and resilience, and their social desires are fulfilled only in a virtual space, which leads to smartphone addiction [7,37]. Parental attitude is a major factor in preventing this negative behavior and its subsequent consequences, such as aggressiveness and depressive symptoms [38,39]. Therefore, parents should focus more on solving this issue since addictive smartphone use among teenagers has expanded very fast during the last decades, and it might become a real global public health problem. Aside from the wide spectrum of psychological problems associated with smartphone addiction, other negative health outcomes were reported, such as musculoskeletal or ocular disorders. Several risk factors were reported to be associated with excessive smartphone use, such as female gender, strict parenting, gaming, social networking, low self-control, and low self-esteem [40,41]. Contrariwise, academic motivation and success were proved to be protective factors in terms of smartphone addiction [40,42].

The COVID-19 pandemic represented a true burden not only for the world-wide communities, but also for each human being. It had an “atomistic–holistic” impact, affecting global, international, national, local, and human levels. Moreover, the negative multiple-domain effect of the pandemic involved global and national economies, health-services-related issues, and social problems. However, the most important impact was probably reflected in poorer interpersonal interaction and human isolation. Although children are commonly less aware of these problems, they were not spared from the negative effects of the COVID-19 pandemic, especially in the context of increased parental stress [43]. Several studies indicated an increased prevalence of sleep disturbances in children and adolescents during the COVID-19 pandemic [43,44,45,46,47]. Aside from parental stress, excessive use of screen media/electronic devices and chronotype were also reported as a trigger of poor sleep quality [46,48,49]. Thus, the effects of the COVID-19 pandemic on sleep patterns are incontestable, but whether the excessive or inappropriate use of smartphones in children is related to the pandemic or not is definitely a major perturbator of human homeostasis in the era of electronics.

Based on all the literature evidence mentioned above, the theoretical framework of our research includes several variables related to smartphone use patterns and their negative psychological and somatic impacts. Thus, the relationship between late-night screen exposure and a decrease in both period and quality of sleep might be explained by the fact that the use of a smartphone before sleep considerably postpones the bedtime hour. Moreover, the engaging content of the smartphone represents an important stimulus for brain activity, thus hindering the transition to sleep. In fact, we can define “a domino” effect since both sleep deprivation and the poor quality of sleep enable a dysfunction of the prefrontal cortex, which is a well-documented modulator of emotions. Unsurprisingly, somatic issues such as ocular or musculoskeletal disorders could be explained by both excessive or late-night screen time, and vicious or prolonged postures, as well as repeated use of the same interphalangeal joints.

Data on smartphone use among Romanian children and adolescents remain limited. A study conducted by Poetar et al. among Romanian adolescents demonstrated an association between problematic smartphone use and mental health-related symptoms; however, the study included only participants aged 13 years and older, supporting the relevance of these associations in the Romanian sociocultural context [50]. Studies involving Romanian children younger than 12 years are lacking, as are studies that simultaneously examine the duration and patterns of smartphone use in relation to sleep disturbances, somatic complaints, and emotional- and anxiety-related symptoms. Therefore, by including both younger children and adolescents, the aim of this study was to examine age-related differences and the impact of smartphone use on Romanian children’s health: sleep disturbances, specific somatic complaints, and emotional- and anxiety-related symptoms. Our hypotheses were that greater daily smartphone use would be associated with: (1) poorer sleep-related outcomes and (2) more somatic complaints and emotional- and anxiety-related symptoms.

## 2. Materials and Methods

### 2.1. Study Sample and Protocol

We conducted a cross-sectional, questionnaire-based study investigating smartphone use among the Romanian pediatric population. Inclusion criteria: age 7–18 years; smartphone ownership. Data collection was conducted between 29 March 2021 and 31 May 2021. The study protocol was approved by the Ethics Committee of George Emil Palade University of Medicine, Pharmacy, Science and Technology Târgu-Mureș, Romania, Approval No. 1313/26.03.2021.

#### 2.1.1. Questionnaire Development and Measures

**Figure 1:**
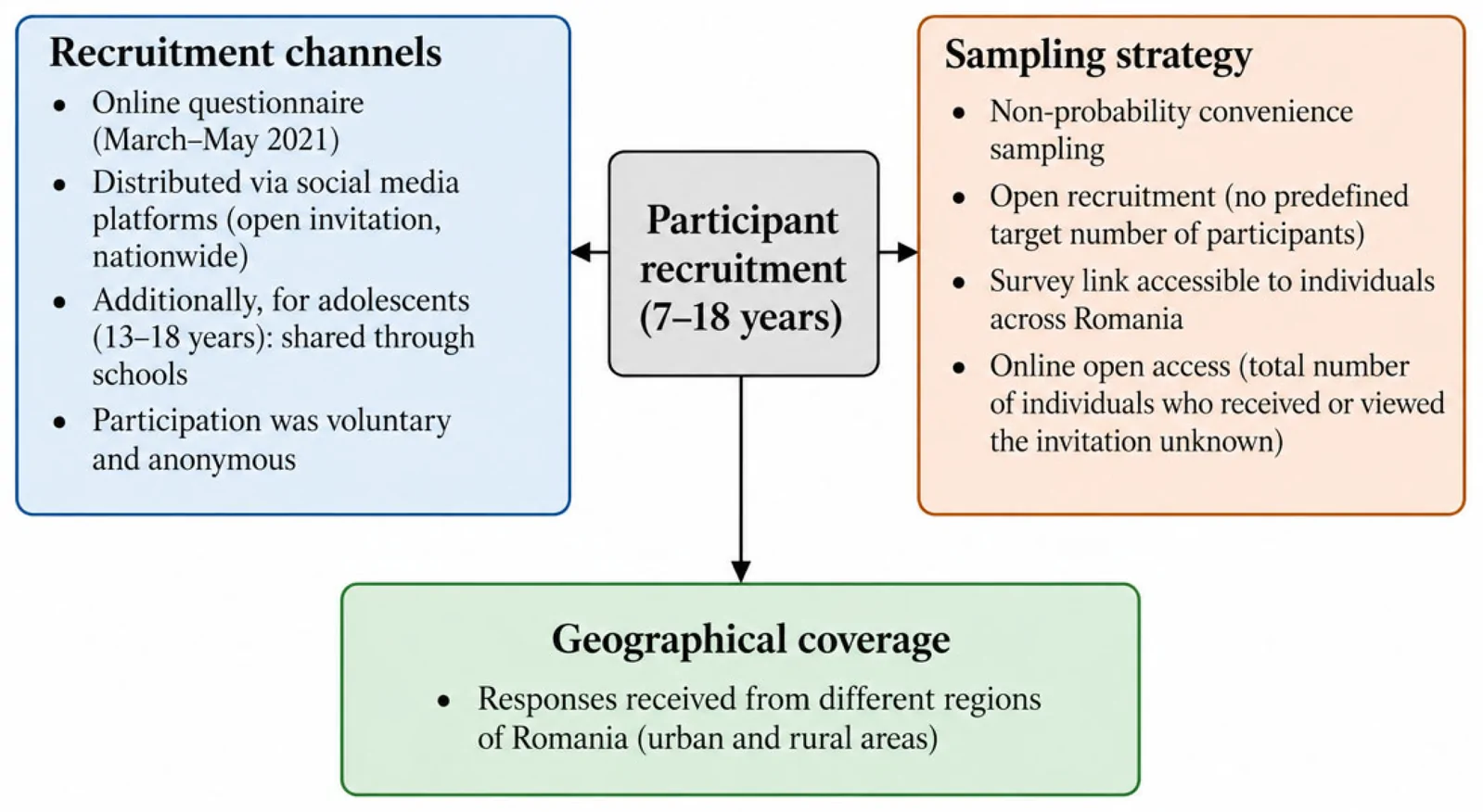
Recruitment process and sampling strategy.

Data were collected using two anonymous online questionnaires developed specifically for the present cross-sectional study. Participation was voluntary, and respondents were recruited using a non-probability convenience sampling approach. The questionnaire was distributed through social media platforms and was accessible to respondents throughout Romania. For adolescents aged 13–18 years, the questionnaire was additionally disseminated through schools in order to facilitate participation in this age group (Figure 1).

No predefined target number of questionnaires or fixed sampling frame was established before recruitment. As the questionnaire was distributed through open online channels and the total number of individuals who received or viewed the invitation could not be determined, a response rate could not be calculated. Responses were received from participants residing in different areas of Romania.

The first questionnaire was designed for children aged 7–12 years (group 1), and was completed with parental assistance: some items were explicitly parent-reported, while other questions were answered by the child with parental assistance. The second questionnaire targeted adolescents aged 13–18 years (group 2) and was completed independently; all information in this age group was self-reported. Both questionnaires comprised 42 questions covering sociodemographic characteristics, smartphone ownership, duration and pattern of use, sleep-related characteristics, somatic complains, and emotional- and anxiety-related experiences. The questionnaires were developed as study-specific data collection instruments and were not intended to function as validated psychometric or diagnostic scales. Consequently, the individual items were analyzed separately, and no composite diagnostic or psychometric scores were calculated. The questionnaire responses should therefore be interpreted as self-reported behavioral characteristics, symptoms, or experiences rather than as validated measures of problematic smartphone use, sleep disorders, depression, anxiety, or other clinical conditions.

#### 2.1.2. General Demographic Data

General demographic information included age, sex, weight, height, place of residence, current educational level, and parents’ highest level of education. Body mass index was calculated and categorized into four groups: underweight, normal weight, overweight, and obesity.

#### 2.1.3. Assessment of Smartphone Use

Smartphone use was assessed as a multidimensional behavior comprising both quantitative characteristics and purposes of use. The questionnaire included several items related to smartphone use, such as smartphone ownership; duration of ownership; purposes and timing of use; daily smartphone use duration for different activities, including interpersonal communication (SMS, smartphone calls, and online communication), social media use, gaming, entertainment (watching movies, listening to music), information seeking, and learning; the perceived importance of smartphone use; and the negative consequences respondents associated with its use. Participants were also asked whether parents and teachers provided guidance regarding appropriate smartphone use. These variables were analyzed as descriptive indicators of different smartphone use activities and were not combined into a composite score. The term “smartphone use” encompasses both the duration of use and the reported purposes of smartphone activity. However, the duration of individual smartphone activities was not assessed separately.

In the present study, excessive smartphone use was considered a descriptive term referring to a high duration of daily smartphone use rather than a diagnosis of smartphone addiction. Daily smartphone use was categorized as <1 h/day, 1–3 h/day, 3–5 h/day, and >5 h/day. Problematic smartphone use or smartphone addiction involves additional behavioral characteristics, particularly impaired control over smartphone use and associated functional impairment, and therefore cannot be determined solely on the basis of daily smartphone use duration.

#### 2.1.4. Assessment of Sleep Characteristics

Sleep characteristics were assessed in terms of both quality and quantity. The average daily sleep duration reported by respondents was compared with the age-specific recommendations of the National Sleep Foundation guidelines. Indicators of poor sleep quality included nocturnal awakenings, nightmares, non-restorative sleep, daytime sleepiness, and daytime napping. Sleep characteristics were assessed using individual questionnaire items rather than a validated pediatric sleep instrument. Accordingly, the sleep-related variables were analyzed as separate indicators, including sleep duration, sleep timing, nocturnal sleep disruption, subjective sleep quality/restoration, and daytime sleepiness, and no composite sleep quality score was calculated. *Sleep duration* was declared by the respondents. The adequacy of sleep duration was evaluated according to age-specific recommendations. Children aged 7–12 years were considered to have an adequate sleep duration when they obtained 9–11 h of sleep per 24 h, whereas for adolescents aged 13–18 years, it was 8–10 h per 24 h, in accordance with the recommendations of the National Sleep Foundation guidelines. *Sleep timing* was the participants reported usual bedtime, which was a separate descriptive variable and was not interpreted as a measure of sleep duration or sleep quality. *Nocturnal sleep disruption* was assessed by asking whether participants woke during the night and, when applicable, whether nocturnal awakenings occurred once or repeatedly. The occurrence of nightmares was assessed separately. Subjective *sleep restoration/quality* was evaluated through the participants’ report of whether they felt refreshed upon awakening. This variable was considered a subjective indicator of restorative sleep rather than a validated measure of overall sleep quality. *Daytime sleepiness* was assessed by asking participants whether they experienced sleepiness during the day and or had daytime naps. This item was analyzed as an indicator of self-reported daytime sleepiness and should not be considered equivalent to a validated daytime sleepiness scale. The *pre-sleep activities* were assessed separately, including smartphone use, reading, television viewing, and other activities before bedtime. *Smartphone use before bedtime* was considered a behavioral exposure potentially related to sleep outcomes rather than a component of sleep quality itself.

#### 2.1.5. Assessment of Health-Related Somatic Symptoms

Questions regarding health-related symptoms covered both somatic and psychological domains. Somatic complains included fatigue, dizziness, headaches, ocular complaints, and pain in the thumb or wrist during or after smartphone use.

#### 2.1.6. Assessment of Emotional- and Anxiety-Related Symptoms

Mental and emotional well-being was explored using individual self-reported questions addressing selected emotional- and anxiety-related experiences. These items were not derived from, and were not intended to constitute, a validated diagnostic instrument for depression or anxiety. Therefore, no composite depression or anxiety score was generated.

Emotional symptoms encompassed indicators such as feelings of sadness, loneliness, loss of interest to play, low self-esteem, perceptions of personal inadequacy, crying without an apparent reason, reduced enjoyment, feelings of inadequacy (“I will never be as good as others”), negative self-evaluation (“I do everything wrong”), self-perceived anxiety, and physiological manifestation of fear. Responses to these items were analyzed individually and were interpreted as self-reported emotional experiences rather than symptoms fulfilling diagnostic criteria for depressive disorders. Anxiety-related symptoms were also assessed using individual questions about excessive worrying, autonomic manifestations of anxiety, fear of being alone at home or sleeping in unfamiliar places, difficulties communicating with strangers, nervousness in public situations, and concerns about being accepted or liked by others. These variables were analyzed separately and were not used to establish a diagnosis of an anxiety disorder. Consequently, throughout the manuscript, the terms “emotional symptoms,” “emotional experiences,” and “anxiety-related symptoms/experiences” are used rather than “depression” or “anxiety disorder,” unless referring to findings from previously published studies.

In addition, respondents were asked about experiences of phantom ringing or phantom vibration.

### 2.2. Statistical Analysis

The statistical analysis included descriptive statistics (frequency, percentage, mean, median, and standard deviation) and inferential statistics. The Shapiro–Wilk test was applied to determine the distribution of the analyzed data series. To compare medians, the Kruskal–Wallis test with Dunn’s Multiple Comparison test was applied, and associations between qualitative variables were assessed using the chi-squared test, or Fisher’s exact test where any expected cell count was below five. Because the variables of interest were ordinal or non-normally distributed, Spearman’s rank correlation coefficient was used to measure the strength and direction of the correlation between the studied variables. All analyses were performed in R version 4.3.3, with a two-sided significance threshold of 0.05.

## 3. Results

**Figure 2:**
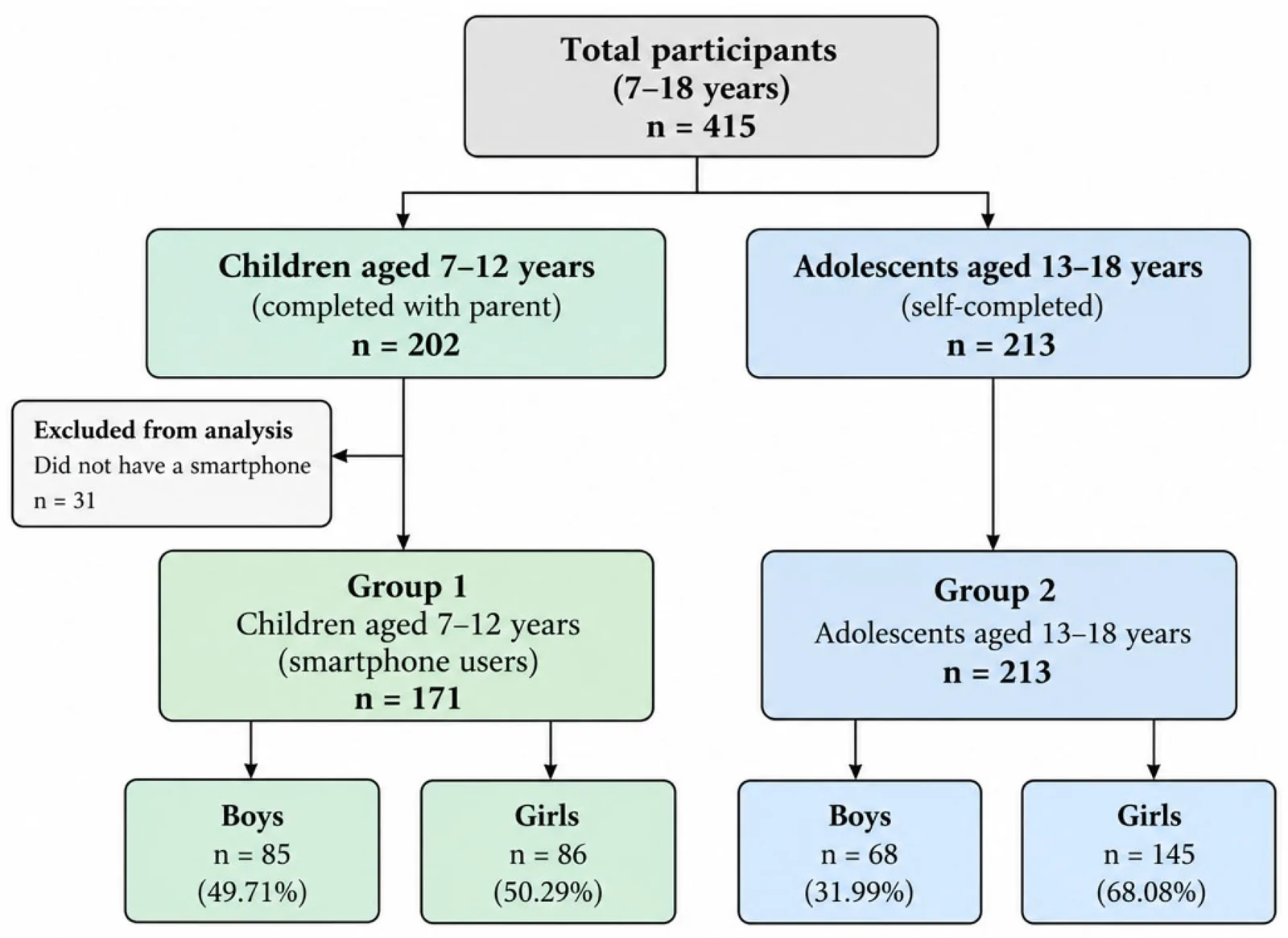
Flow diagram of participant selection and study groups.

A total of 415 children and adolescents were included in the study: 202 children aged 7–12 years (group 1) and 213 adolescents aged 13–18 years (group 2). All respondents in group 2 reported owning a smartphone. In group 1, 3.46% (n = 7) owned a conventional mobile phone, 84.65% (n = 171) owned a smartphone, and 11.88% (n = 24) did not own a personal mobile phone. As the present study focused on smartphone users, participants without a personal smartphone were excluded from further analyses (those with conventional phones or without any phones, n = 31), resulting in a final sample of 171 children in group 1. Thus, the final analytical sample for analyses concerning smartphone use consisted of 384 participants (171 in group 1 and 213 in group 2) (Figure 2).

The mean age of participants in group 1 was 10.34 ± 1.653 years. Girls represented 50.29% of the sample, while boys accounted for 49.71%. More than half of the participants (53.22%) were enrolled in primary school, whereas 46.78% were enrolled in a gymnasium program. The majority resided in urban areas (57.31%). In group 2, the mean age was 15.73 ± 1.590 years. Females comprised 68.08% of the sample and males 31.92%. Most participants (67.14%) were enrolled in high school, 27.70% were enrolled in a gymnasium program, and only 5.16% were students in vocational educational programs. Similar to group 1, most adolescents lived in urban areas (55.87%). See the descriptive analysis data in Table 1, Table 2, Table 3 and Table 4.

Regarding parental educational attainment, 42.69% of mothers and 35.67% of fathers in group 1 had pursued education beyond high school. In group 2, only 35.21% of mothers and 28.17% of fathers held a university degree. A statistically significant association was identified between parental educational level, and daily smartphone use among adolescents in group 2. Teenagers whose parents had attained a university degree were significantly more likely to spend longer periods using their smartphones compared with those whose parents had not completed higher education (OR = 3.268, 95% CI [1.74–5.93], *p* = 0.0001).

### 3.1. Smartphone Use

With regard to the duration of smartphone ownership, nearly half of the adolescents in group 2 (46.48%) had owned a smartphone for more than 5 years. In contrast, the majority of children in group 1 (46.78) had owned their smartphones for 1–3 years, whereas only 3.51% reported smartphone ownership exceeding five years.

When asked about the reason for obtaining a smartphone, the most frequently reported motive in both age groups was maintaining communication with family members (67.84% in group 1 and 90.61% in group 2). This was followed by keeping in touch with friends (28.65%—group 1 and 60.09%—group 2), educational purpose (40.35%—group 1 and 35.68%—group 2), or entertainment or gaming (36.84%—group 1 and 35.68%—group 2). In both groups, educational purposes were significantly associated with more recent smartphone acquisition, specifically ownership for less than one year or for 1–3 years (group 1—OR = 3.351, 95% CI [1.38, 8.78], *p* = 0.0105 and group 2—OR = 4.091, 95% CI [1.85, 8.73], *p* = 0.0003). The finding likely reflects the widespread implementation of online learning during the study period (COVID-19 pandemic).

Most participants in both age groups reported spending more time on their smartphones during weekdays than on weekends. Increased weekday use was reported by 64.33% of children in group 1 and 68.54% of adolescents in group 2, whereas during weekends, increased weekday use was reported by 35.67% and 31.46%, respectively.

Regarding daily smartphone use, approximately half of the children in group 1 spent less than three hours per day using their devices, with 8.77% reporting less than one hour and 40.35% reporting 1–3 h of daily use. In contrast, approximately two-thirds of adolescents in group 2 used their device for more than three hours per day, including 40.85% who reported 3–5 h of use and 26.76% who reported more than five hours daily.

A statistically significant positive correlation was observed between duration of smartphone ownership and daily smartphone use in both age groups, indicating that participants who had owned a smartphone for a longer period tended to spend more time using it each day (group 1: r = 0.3110, 95% CI [0.16–0.44], *p* < 0.0001; group 2: r = 0.2030, 95% CI [0.06–0.32], *p* = 0.0029) (Table 5).

Following Dunn’s Multiple Comparison test, age was found to be significantly associated with the duration of daily smartphone use in group 1. Specifically, older children were significantly more likely to use their smartphones for longer periods with significant differences observed between the categories of <1 h/day, 1–3 h/day, and 3–5 h/day compared with >5 h/day (*p* = 0.0003, *p* < 0.0001, and *p* = 0.0005, respectively) (Table 6). In contrast, age was not significantly associated with daily smartphone use among adolescents in group 2 (*p* = 0.3670, *p* = 0.0990, and *p* = 0.1408) (Table 7).

With respect to gender differences, no significant association was identified between sex and the number of hours spent using smartphones per day in group 1 (*p* = 0.7163). However, in group 2, girls reported significantly longer daily smartphone use than boys, with a median usage time of four hours per day compared with two hours per day among boys (*p* < 0.0001).

Participants in both groups primarily viewed smartphones as tools for communication and information. Smartphone use for communication was reported by 96.71% of adolescents in group 2 and 70.76% of children in group 1, while use for obtaining information was reported by 81.22% in group 1 and 61.99% in group 2. In addition, smartphones were used for entertainment by 64.79% of the participants in group 2 and 51.46% in group 1. In group 1, girls were significantly more likely than boys to use smartphones for communication (RR = 1.357, 95% CI [1.11–1.658], *p* = 0.0025), but this difference was not significant in group 2 (RR = 1.017, 95% CI [0.96–1.078], *p* = 0.6823).

When asked what they use their smartphones for, the majority of participants (81.87%) in group 1 chose learning, followed by playing games (75.29%), making phone calls (52.63%), sending messages or online chatting (52.63%), listening to music (47.37%), and browsing social media platforms (29.82%).

In group 2, the most frequently reported smartphone activities were online chatting (92.96%), browsing social media platforms (89.20%), and learning (85.44%), but also making calls (82.63%) and listening to music (80.75%), whereas gaming was less common (51.47%).

In both groups, boys were more likely than girls to use smartphones for gaming (75.29% in group 1 and 51.47% in group 2, respectively). However, boys had a higher odd of using their smartphones for gaming compared with girls (aOR = 3.55, 95% CI [1.83–7.09]).

**Figure 3:**
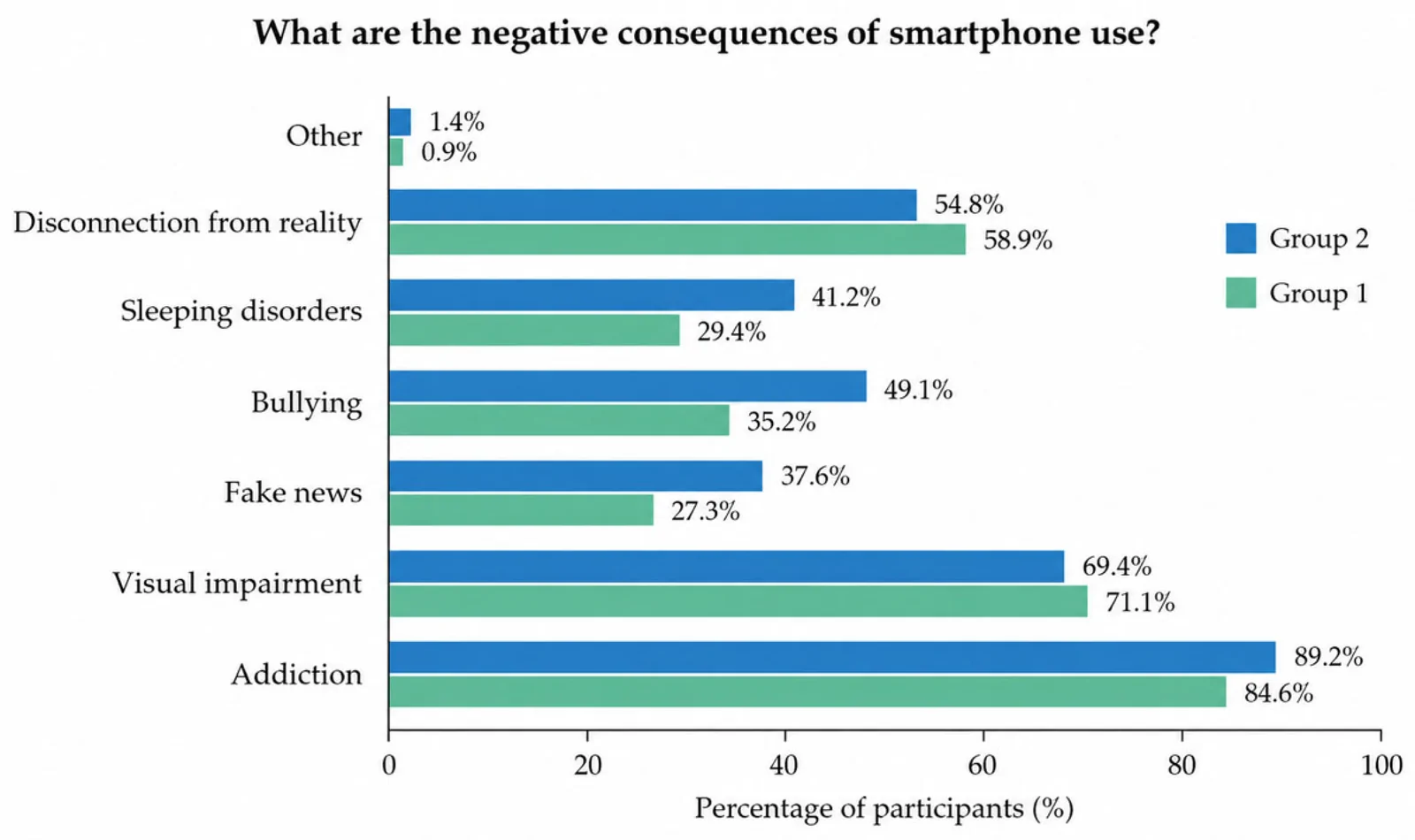
Negative consequences of smartphone use (%).

The majority of subjects in both groups perceived their smartphone use as excessive, with 74.27% of children in group 1 and 72.77% in group 2. Furthermore, an even greater proportion (87.13% in group 1 and 80.29% in group 2) reported perceiving negative consequences associated with smartphone use. Participants were also asked to identify the potential negative consequences they associated with smartphone use (Figure 3). In both groups, smartphone addiction was the most frequently reported adverse outcome, which was mentioned by 84.79% of children in group 1 and 89.20% of adolescents in group 2, followed by visual impairment (71.92% in group 1 and 69.95% in group 2) and disconnection from reality (57.89% in group 1 and 54.46% in group 2, respectively).

Sleep disturbances, cyberbullying, and exposure to fear-inducing or alarming content were each reported by approximately 30% of respondents in group 1 and around 40% of those in group 2. Additional possible adverse outcomes included: exposure to age-inappropriate content, reduced time spent outdoors, decreased level of physical activity, poor posture, and wasting time.

### 3.2. Sleeping Time and Sleep Disorders

With regard to sleep duration, comparison with the age-specific recommendations of the National Sleep Foundation (NSF) revealed that almost half of respondents in both age groups (47.37% of group 1 and 53.99% of group 2) did not achieve the recommended amount of sleep. In group 1, sleeping time was 8.804 ± 0.9462 SD hours/day, while in group 2 it was 7.585 ± 1.073 SD hours/day. According to the results detailed in Table 8, we noticed that sleep duration significantly decreased among both groups as the phone ownership period increased.

Moreover, the study highlighted that the more time children and adolescents spend on their smartphones, the later they go to bed (group1: r = 0.1611, 95% CI [0.00–0.30], *p* = 0.0353; group 2: r = 0.3027, 95% CI [0.17–0.42], *p* < 0.0001). Not only bedtime hour but also sleep duration was modified by the length of time they spent on their phones. There was a statistically significant difference between the medians of the sleep duration values of the subjects who used phones for less than 3 h per day versus more than 3 h/day in group 2, but the difference was not significant for group 1 (Table 9). Thus, teenagers had a 2 times greater probability of inadequate sleep duration if they used the phone more than 3 h per day (OR = 2.052, 95% CI [1.07–3.80], *p* = 0.024).

**Figure 4:**
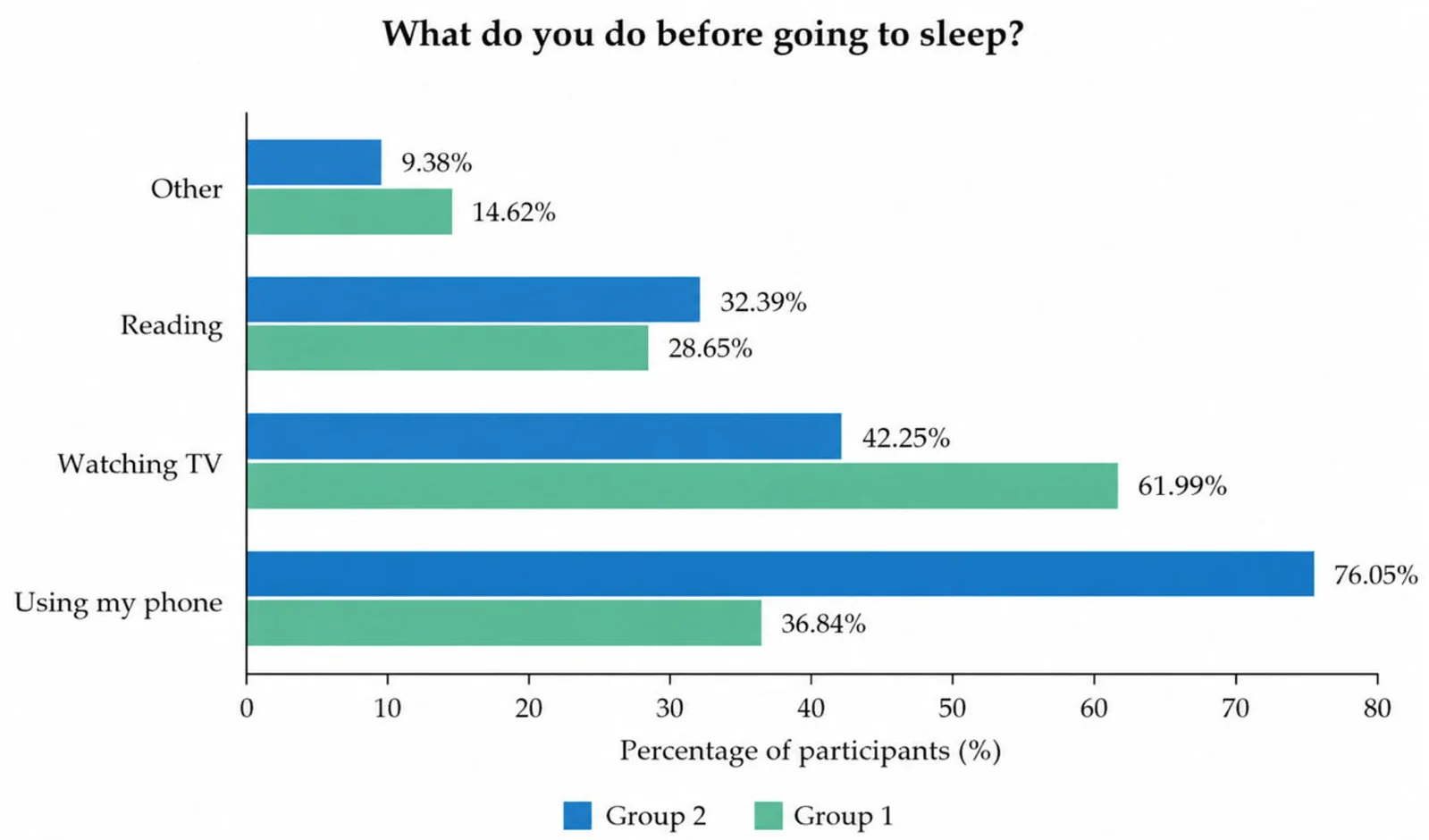
Activity before going to bed (%).

One third (36.84%) of group 1 and 76.05% of group 2 used their phone before going to bed (Figure 4).

A statistically significant association was found between smartphone use before bedtime and insufficient sleep duration in group 1 (OR = 2.842, 95% CI [1.51–5.47], *p* = 0.009). Regarding the quality of sleep, we found that 27.49% of the subjects in group 1 and 44.61% in group 2 woke up during the night, most of them once (85.11%—group 1 and 69.47%—group 2). The association between waking up at night and smartphone use before bedtime was not statistically significant (group 2 *p* = 0.506, group 1 *p* = 0.103). Nightmares were reported by 37.43% in group 1 and 52.58% in group 2. Moreover, more than half of the respondents in both groups (54.97% in group 1 and 82.16% in group 2) experience non-restorative sleep and not feeling refreshed upon waking in the morning. This complaint was significantly more common in those who reported using the phone before bedtime (group 1: OR = 3.382, 95% CI [1.75–6.77], *p* = 0.004; group 2: OR = 2.91, 95% CI [1.37–6.11], *p* = 0.024). Duration of smartphone use during the daytime was associated with poorer quality of sleep since a statistically significant association was observed between daily duration of smartphone use and feeling refreshed upon waking in the morning in both groups (group 1—*p* = 0.0170, group 2—*p* = 0.0001). In teenagers (group 2), the presence of at least two of the three sleep quality parameters examined (waking up during sleep, bad dreams, or lack of restfulness on waking) was significantly associated with the number of hours of daily smartphone use (more than 3 h daily phone use: OR = 3.679, 95% CI [2.02, 6.60], *p* < 0.0001; more than 5 h: OR = 2.373, 95% CI [1.22, 4.71], *p* = 0.0115). Likewise, a significant correlation was found among the same group regarding smartphone use before falling asleep and the presence of at least two complaints of poor sleep quality (OR = 1.969, 95% CI [1.03, 3.75], *p* = 0.0357).

Daytime sleepiness was reported by 46.78% in group 1 and 79.34% in group 2; while 12.86% in group 1 and 23.47% in group 2 confirmed sleeping during the day. In group 2, daytime sleepiness was associated with smartphone use before sleep (crude OR = 3.25, 95% CI 1.59–6.63) and with more than three hours of daily use (crude OR = 3.74, 95% CI 1.88–7.44); after adjustment, these estimates were attenuated to aOR = 1.84 (95% CI 0.79–4.20) and aOR = 2.35 (95% CI 1.10–5.04), respectively, and neither retained significance after correction for multiple testing.

After adjustment and correction for multiple testing, four associations remained statistically supported. In group 1, smartphone use before bedtime was associated with insufficient sleep duration (aOR = 2.58, 95% CI 1.27–5.35, q = 0.041) and with non-restorative sleep (aOR = 2.92, 95% CI 1.43–6.15, q = 0.041). In group 2, daily use exceeding three hours was associated with insufficient sleep duration (aOR = 2.50, 95% CI 1.32–4.83, q = 0.041) and with disturbing dreams (aOR = 2.43, 95% CI 1.26–4.75, q = 0.041). Notably, the dominant exposure differed by age group: in younger children, the timing of use—specifically use before bedtime—was the operative factor, whereas in adolescents, the operative factor was the total daily duration of use. Boys had higher odds of using their smartphone for gaming than girls in both groups (group 1: aOR = 3.55, 95% CI 1.83–7.09, q < 0.001; group 2: aOR = 2.02, 95% CI 1.08–3.82, q = 0.028).

A graded relationship between the amount of daily smartphone use and several outcomes was evident across the four exposure categories, particularly among adolescents, where the prevalence of disturbing dreams rose from 0% among those using their device for less than one hour per day to 70.2% among those using it for more than five hours, and the prevalence of fatigue rose from 14.3% to 52.6% across the same categories. Tests for departure from linearity were non-significant for all eighteen outcome–group combinations examined (all *p* > 0.08), indicating that the dichotomization at three hours does not distort the underlying dose–response relationship.

### 3.3. Somatic Complaints

**Figure 5:**
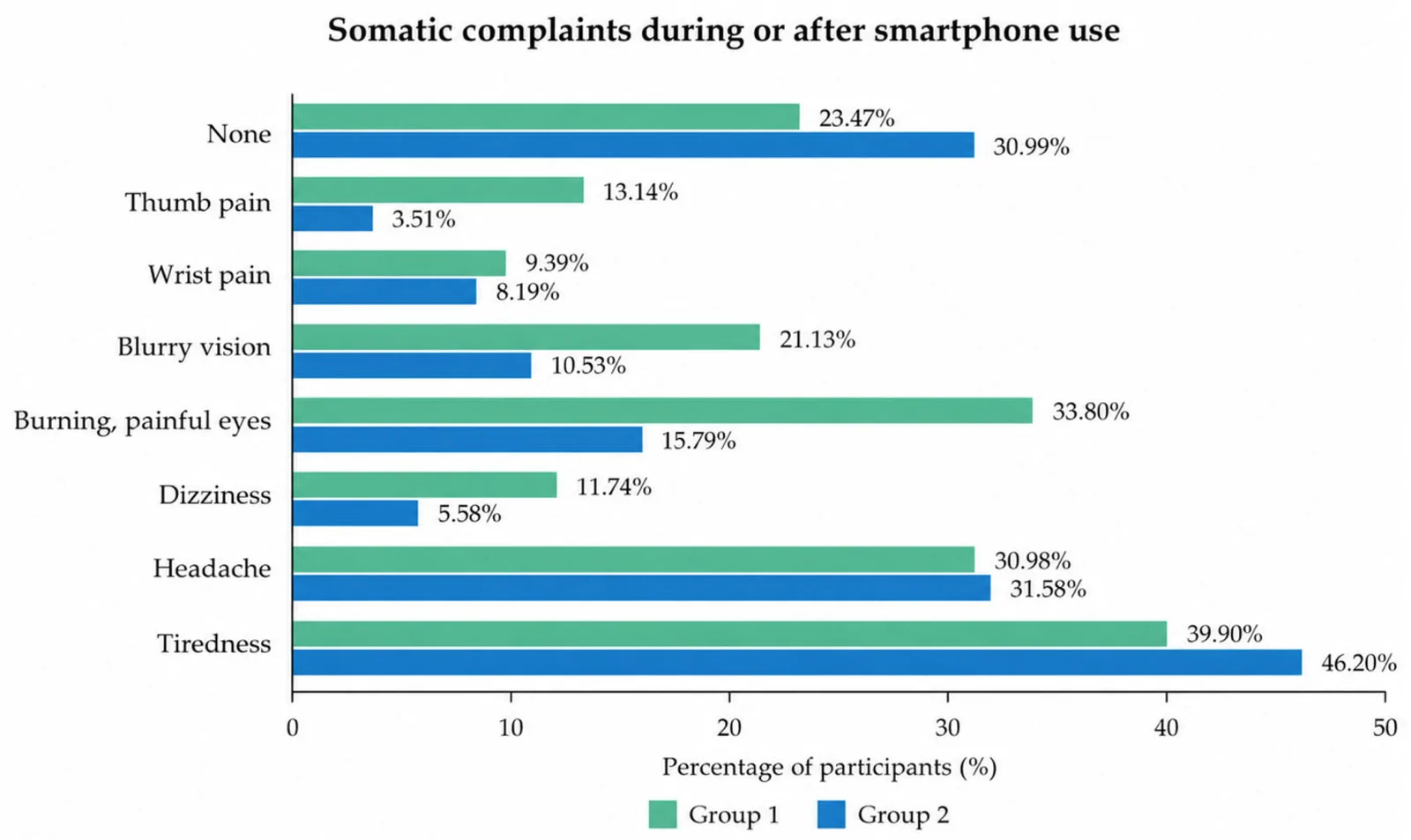
Somatic complaints during or after smartphone use (%).

During or after smartphone use, 69.01% of children in group 1 and 76.53% of adolescents in group 2 experienced several physical symptoms. The most common symptom reported by group 1 was tiredness (46.20%), followed by headache (31.58%) and ocular symptoms (pain and burning sensation 15.79%, blurred vision 10.53%). In group 2, fatigue and tiredness also ranked first (39.91%), followed by pain and burning sensation in the eye (33.80%), headache (30.99%), and blurred vision (21.13%). Dizziness, wrist, and thumb pain were reported less frequently. Nevertheless, all three symptoms were more common in group 2 (Figure 5).

In unadjusted analyses, the presence of one or more physical complaints (tiredness, headache) was associated with daily smartphone use exceeding three hours in group 2 (crude OR = 2.17, 95% CI 1.13–4.16), and fatigue was more frequent among heavier smartphone users in both groups (group 1: crude OR = 2.10, 95% CI 1.14–3.88; group 2: crude OR = 2.47, 95% CI 1.31–4.63). None of the associations within the somatic domain remained significant after correction for multiple testing (all q > 0.10), and all were further attenuated after adjustment for age, sex, residence, sleep duration, and bedtime smartphone use. These findings should therefore be regarded as exploratory and require confirmation in adequately powered studies.

Based on body mass index (BMI), we noticed that 16.96% of children in group 1 were overweight and 12.28% obese, while in group 2, 10.33% were overweight and 3.29% were obese. In the younger group, we found a statistically significant correlation between the duration of daily phone use and BMI (Spearman correlation coefficient, (r = 0.3882, 95% CI [0.24–0.51], *p* < 0.0001), meaning that the increase in BMI is associated with the increase in the daily hours spent using a phone, especially when the number of hours passes 3 h (Table 10 and Table 11).

### 3.4. Psychological Complaints

**Figure 6:**
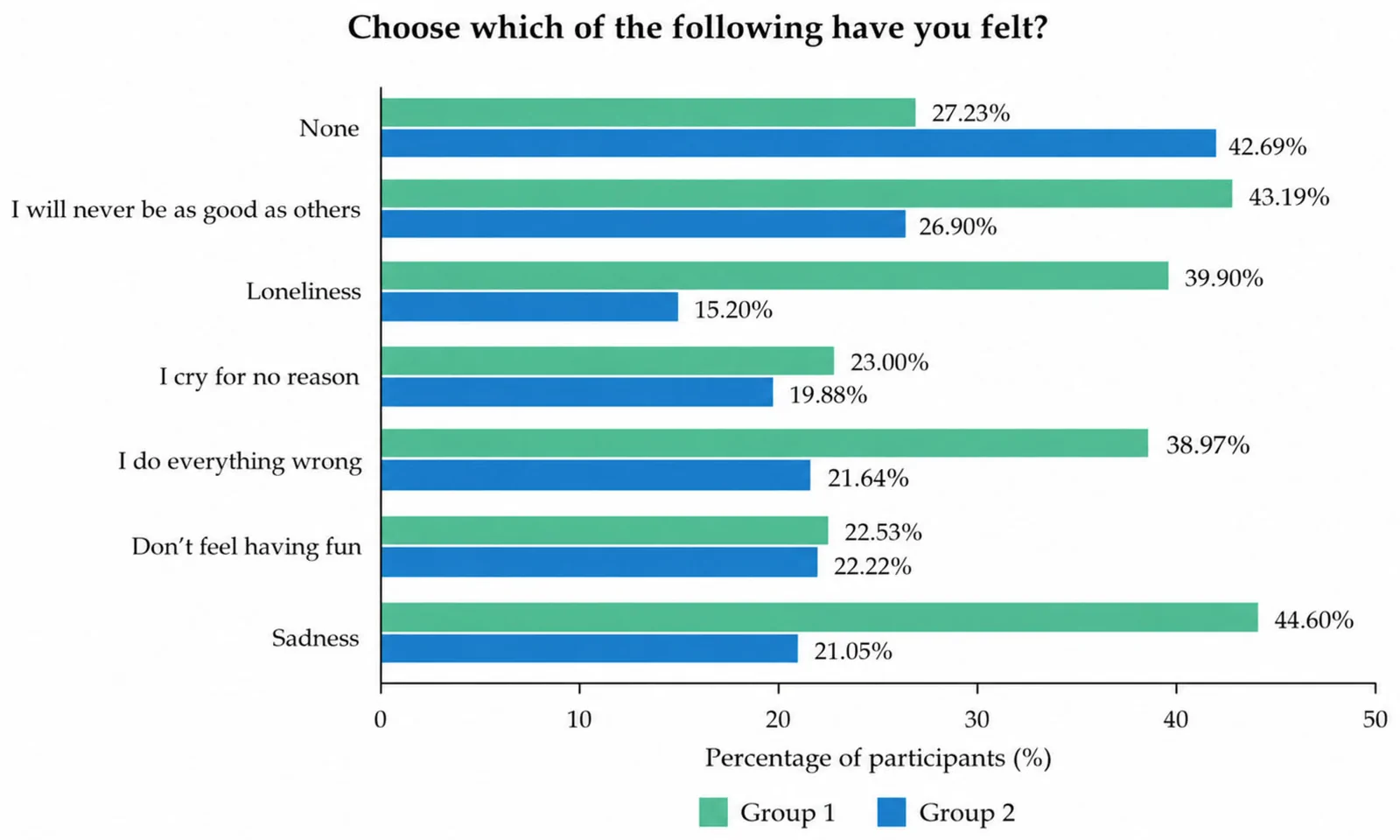
Self-reported emotional symptoms/experiences (%).

Psychological symptoms included auditory and tactile hallucinations, self-reported emotional symptoms/experiences (formulated in the questionnaire as depressive complaints), and anxiety-related symptoms/experiences (formulated in the questionnaires as anxiety symptoms). A total of 11.11% in group 1 experienced phantom vibration and 16.96% phantom ringing, with no significant differences between genders. In group 2, phantom vibration appeared in 38.97%, while phantom ringing in 34.27% with a significantly higher probability compared to group 1 (OR = 3.922, 95% CI [2.45, 6.25], *p* < 0.0001), and the risk of experiencing auditory hallucinations was significantly higher among females (RR = 1.813 95% CI: 1.112–2.958 *p* = 0.0156). Even more concerning, less than half of subjects included in this study, 42.69% in group 1 and 27.23% in group 2, were free of self-reported emotional symptoms/experiences (Figure 6).

There was a statistically significant association between smartphone use before bedtime and the association of self-reported emotional symptoms/experiences (formulated as depressive symptoms in the questionnaire) in both groups (group 1 *p* = 0.018; group 2 *p* = 0.045). In addition, for group 2, smartphone use for more than 3 h/day was associated with the presence of one or more symptoms (OR = 3.187, 95% CI [1.72–6.00], *p* = 0.0002).

In terms of anxiety-related symptoms/experiences, 93.57% of group 1 and 98.12% of group 2 reported experiencing at least one of the listed complaints. In group 1, 54.97% indicated that they were anxious, 57.31% became anxious when they did something in the presence of others, and 46.20% worried whether people like them. Likewise, in group 2, 70.90% of adolescents said they were anxious, 67.61% were nervous when they had to do something in the presence of others, 59.16% were anxious about whether others liked them, and tachycardia was associated in 38.57% of adolescents as a vegetative symptom. No significant association was found—neither in group 1 (*p* = 0.837) or group 2 (*p* = 0.692)—between using the smartphone for more than 3 h per day and the occurrence of anxiety-related symptoms/experiences or smartphone use before falling asleep and anxiety-related symptoms/experiences (group 1 *p* = 1.000, group 2 *p* = 1.000).

### 3.5. Multivariable Analysis

After adjustment and correction for multiple testing, four associations remained statistically supported (Table 12). In group 1, smartphone use before bedtime was associated with insufficient sleep duration (aOR = 2.58, 95% CI 1.27–5.35, q = 0.041) and with non-restorative sleep (aOR = 2.92, 95% CI 1.43–6.15, q = 0.041). In group 2, daily use exceeding three hours was associated with insufficient sleep duration (aOR = 2.50, 95% CI 1.32–4.83, q = 0.041) and disturbing dreams (aOR = 2.43, 95% CI 1.26–4.75, q = 0.041). Notably, the dominant exposure differed by age group: in younger children, the timing of use—specifically use before bedtime—was the operative factor, whereas in adolescents, the operative factor was the total daily duration of use. Boys had higher odds of using their smartphone for gaming than girls in both groups (group 1: aOR = 3.55, 95% CI 1.83–7.09, q < 0.001; group 2: aOR = 2.02, 95% CI 1.08–3.82, q = 0.028). Of the eighty tests performed, twenty-two remained significant after control of the false discovery rate within the families.

**Figure 7:**
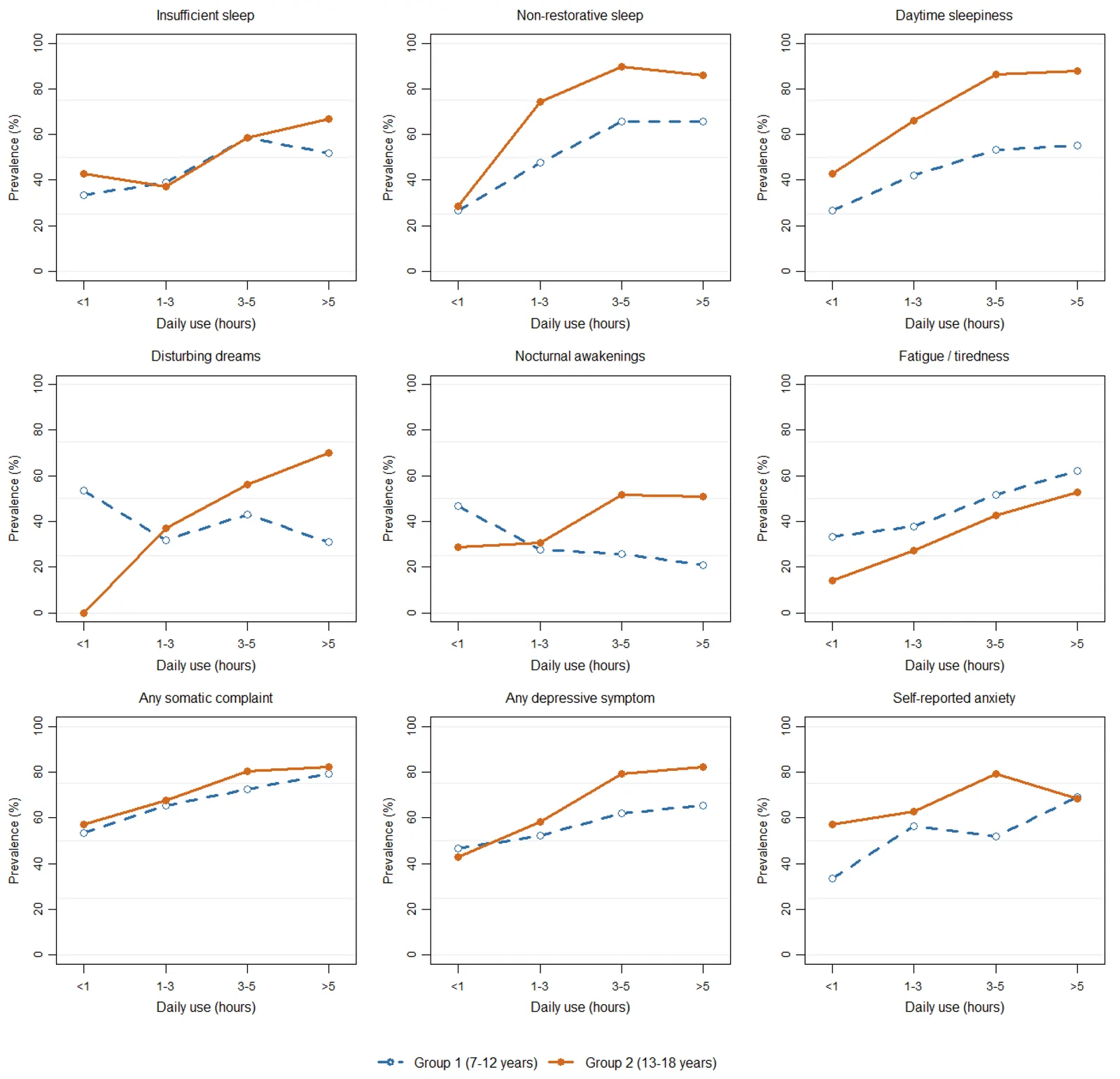
Prevalence of each outcome across the four categories of daily smartphone use, by age group. Group 1 (7–12 years): n = 15, 69, 58, and 29 across the four categories; group 2 (13–18 years): n = 7, 62, 87, and 57. Estimates for the lowest exposure category in group 2 are based on seven participants and should be interpreted with caution. Tests for trend and for departure from linearity are given in Table 13.

A graded relationship between the amount of daily smartphone use and several outcomes was evident across the four exposure categories, particularly among adolescents, where the prevalence of disturbing dreams rose from 0% among those using their device for less than one hour per day to 70.2% among those using it for more than five hours, and the prevalence of fatigue increased from 14.3% to 52.6% across the same categories (Table 13, Figure 7). Tests for departure from linearity were non-significant for all eighteen outcome–group combinations examined (all *p* > 0.08), indicating that the dichotomization at three hours does not distort the underlying dose–response relationship.

## 4. Discussion

A real and comprehensive picture of how different age groups of Romanian children use their smartphones and how the use of these devices leads to health problems is currently lacking. The findings of this study were meant to fill this gap. Most of the children included in both groups (7–12 years of age—group 1, and 13–18 years of age—group 2) originated from the urban area. In terms of gender, we noticed that female teenagers spent significantly more time on their phones when compared to male teenagers (*p* < 0.0001). Importantly, total smartphone use duration should not be interpreted as a homogeneous exposure. Smartphones support a wide range of activities, including interpersonal communication, social media use, gaming, entertainment, information seeking, and educational activities, which may differ in their associations with sleep, physical complaints, and emotional well-being. Although our questionnaire captured the purposes for which participants used their smartphones, it did not assess the duration or frequency of each specific activity. Consequently, the associations observed for total smartphone use duration cannot be attributed to a particular type of smartphone activity. A recent study underlined that female adolescents have a greater risk of experiencing sleep problems, highlighting a significant correlation between this complaint and excessive daily smartphone use [5,51]. Moreover, the study of Claesdotter-Knutsson found that feelings like anxiety, sadness, and worry trigger excessive smartphone use among female teenagers [52]. Contrariwise, our findings revealed that males included in our study were more likely to use their phone for gaming, independent of their age, with a concerning higher risk in younger ages. Similar findings were also reported by previous research [53,54,55]. Almost two thirds of the participants included in this study, regardless of their age, acknowledged both the excessive use of their phone every day and their “addiction” (formulation used in questionnaire) to it. Aside from excessive smartphone use, other adverse effects of excessive daily phone use associated in our respondents were visual impairment, disconnection from reality, sleep problems, cyberbullying, and scaremongering.

Eye and vision problems related to daily excessive screen use is a common health problem nowadays. According to American optometrists, 14.25% of the patients visiting the optometry clinic were diagnosed with computer vision syndrome defined by the American Optometric Association as “a complex of eye and vision problems related to near work experienced during computer use” [56]. This syndrome can be extrapolated to screen vision syndrome, or perhaps the symptoms caused by excessive phone screen time could be even more severe, taking into account that the sizes of a phone screen are considerably smaller than that of a computer, and that when using a phone, we require a shorter distance from the screen. A study performed on medical students highlighted that 95% of the participants experienced at least one symptom of computer vision syndrome [57,58]. In our study, ocular symptoms were associated as chief complaints in both groups as follows: in more than 10% of the children aged between 7 and 12 years (group 1), smartphone use was associated with pain and burning sensation (15.79%), as well as blurred vision (10.53%), while the frequency of these symptoms considerably increased among teenagers, 33.8% of them experienced pain and burning sensation in the eye, and 21.13% reported blurred vision.

Upper limb neuromusculoskeletal dysfunction is another major health condition related to excessive smartphone use. Thus, impairment of the deep neck flexors due to vicious posture, such as forward head posture or poking chin posture, was associated with excessive smartphone use when seated, resulting in persistent tissue overloading, and eventually neck pain [59,60]. Similarly, hand function impairment is commonly found among heavy smartphone users consisting in dysfunction of hand and pinch grip [61,62]. In our study, although wrist and thumb pain were not among the most frequent associated symptoms, they were more commonly reported by teenagers, suggesting a possible time-dependent relation between hand impairment and phone use. Nevertheless, headache was the main complaint in both groups of our study, with this symptom being present in more than a quarter of the respondents in each group (group 1—31.58%, group 2—30.99%). Although the presence of these symptoms in young ages remains alarming, after correction, none of the associations in terms of somatic symptoms preserved its significance.

It is well documented that sleep is an indispensable need of the human being to maintain its homeostasis. However, in terms of individuals below the age of 18 years, sleep is even more important since it enables proper physical growth and psychic development, but at the same time it decreases morbidity related to the cardiovascular, hormonal, and immune systems [63,64,65,66]. Sleep duration is not the only factor that influences the quality of sleep. Sleep continuity and efficiency, alertness or sleepiness, and timing and satisfaction or quality are other factors [51,67,68,69]. Studies assessing sleep quality pointed out that characteristics like age, gender, and lifestyle habits defined as addiction, but also socioeconomic, cultural, and psychosocial factors, strongly influence sleep quality, which was indicated to decrease with age [70,71,72,73]. An interesting finding of our study that might be indirectly related to the aforementioned statement is that in terms of group 1, we identified a significant increase in daily phone use with age, suggesting that it might also contribute to the increased frequency of poor sleep quality in older ages.

Most of the available studies so far report alarming rates of poor sleep quality in children and adolescents [5,13,74], although the range varies between 9.8% and 60.4% depending on the geographic area [70,75,76]. Our study also revealed that almost half of the children included in group 1 (47.37%, 7–12 years of age) and more than half of those included in group 2 (53.99%, 13–18 years of age) did not get enough sleep. The findings of our study are especially alarming in terms of younger children, suggesting a remarkable increased rate of insufficient sleep duration in Romanian children below the age of 12 years. Moreover, our study revealed that increased daily phone use was associated not only with poorer sleep duration, but was also related to bedtime hour since we noticed that the more time children and adolescents spend on their smartphones, the later they go to bed (group1: r = 0.1611, 95% CI [0.00–0.30], *p* = 0.0353; group 2: r = 0.3027, 95% CI [0.17–0.42], *p* < 0.0001). In addition, the teenagers included in this study had a two times greater probability of inadequate sleep duration if they used their phone more than three hours per day (OR = 2.052, 95% CI [1.07–3.80], *p* = 0.024). Regarding sleep quality, our study revealed associations, either significant or not, of both phone use before sleep and excessive daily phone use on several parameter’s indicative of poor sleep quality in both groups, such as nighttime awakenings, nightmares, or awakening fatigue. It is worth mentioning that among the teenagers included in our study, we found significant associations between the two variables for at least two of the three sleep quality parameters examined (waking up during sleep, bad dreams, or lack of restfulness on waking), while in group 1, we found a significant association only between the amount of time spent daily using a phone and "feeling not rested when waking up". Although daytime sleepiness was a common symptom reported by both age groups included in this study (46.78% in group 1 and 79.34% in group 2), we found this symptom to be associated with smartphone use before bedtime and increased daily use only in teenagers. Similar to our findings, the study of Kim et al. also indicated that teenagers who felt unsatisfied with their sleep present a significantly greater period of daily smartphone use and scored higher on smartphone addiction scales when compared to those who were satisfied with their sleep [77]. Another study performed on Indian teenagers and young adults concluded that increased daily phone screen time is associated with a higher chance of experiencing sleeping problems [69]. Thus, there is no doubt that increased daily phone has a significant negative influence on sleep quality. Moreover, poor sleep quality has been consistently associated with depressive symptoms, fatigue, reduced school engagement, impaired daily functioning, and compromised immune function [23,78,79]. An interesting finding of our research was the difference between the factor that influences both sleep duration and quality in younger children when compared to teenagers. Thus, the timing of use (before bedtime) was the main negative factor involved in poor sleep quality and insufficient sleep, while in teenagers, we noticed total daily use to be more likely involved in decreasing both the quality and amount of sleep.

Previous studies associated excessive smartphone use with depression and anxiety [26,80,81]. Further research emphasized a reciprocal relationship between smartphone addiction and depression, suggesting that the first one is commonly characterized by the latter, and that depression worsens smartphone addiction [37,79,82]. The fact that depression and smartphone addiction resemble the ying–yang concept during adolescence is no longer a paradigm. Our findings are consistent with previous studies, since less than half of subjects included in this study, 42.69% in group 1 and 27.23% in group 2, were free of emotional symptoms/experiences suggestive for depression. Thus, the most alarming finding of our study is that these emotional symptoms/experiences suggestive for depression symptoms tend to be associated predominantly with younger children. Moreover, we noticed that phone use before bedtime was significantly associated with the onset of emotional symptoms/experiences suggestive for depression, regardless of the child’s age. In terms of anxiety, we found this association only for teenagers, suggesting that increased awareness of acts and their consequences present in older ages may increase anxiety symptoms triggered by excessive phone use. It is not uncommon for depression and anxiety to occur together. According to recent studies, there is an even deeper relationship between smartphone use and anxiety, implying a positive correlation between excessive and problematic smartphone use and poor sleep quality, as well as between sleep quality and anxiety [80,83]. Thus, sleep quality might be considered a mediator between the impact of smartphone use and anxiety symptoms, especially in individuals experiencing sleep problems [83]. Extremely concerning regarding our findings was that almost all participants included in our study (group 1—93.57%, group 2—98.12%) reported at least one symptom from the anxiety-related symptoms/experiences. Furthermore, a recent study confirmed a causal relationship between daily phone use and mental health, underlining that a 3-week reduction in daily phone screen time improved sleep quality, depressive symptoms, stress, and well-being [84].

Smartphone addiction was associated with other addictions, such as smoking, alcohol abuse, and the consumption of other substances [53,85]. Among these, smoking is a well-known public health problem, especially in young people [86]. But smartphone addiction is an emerging global public health problem with even greater long-term consequences than smoking, since it has a major long-term negative impact on individuals due to its occurrence at an earlier age. The implementation of public health strategies meant to reduce smartphone use especially, but not solely, in young children, is crucial for maintaining the physical and mental health of people worldwide.

No consensus threshold for excessive daily smartphone use exists in this literature, and published studies have adopted cut-points ranging from two to five hours per day [87,88,89]. A recent dose–response meta-analysis of seventeen observational studies found the association between daily smartphone use and poor sleep quality to be essentially linear, with no natural inflection point [90], indicating that any dichotomization is a matter of convention rather than of an underlying discontinuity. We retained the three-hour threshold because it corresponds to the upper exposure category employed by Maurya et al. [69], which allows for the direct comparison of our estimates with theirs; the prevalence of each outcome across all four exposure categories is nevertheless reported in Table 13, where tests for departure from linearity were non-significant throughout.

The theoretical value of our research is mainly limited to generally confirming the existing theories, except for the influence of smartphone use on chronotype, which was different depending on the respondent’s age. Thus, our study highlighted that in younger children, the chronotype is affected by the use of smartphones before bedtime, while in teenagers, it is rather impaired by the total daily screen time. Conversely, the practical contributions of our research are incontestable and should represent the cornerstone for national health policies regarding the use of electronic devices in pediatric ages, which should involve tailored recommendations for age and gender, taking into account findings like increased frequency of smartphone use for gaming in boys, the fact that female gender is more prone to an increased total screen time, and even the different impacts of smartphone use on children’s health outcomes.

The limitations of this study consist primarily of the relatively small number of respondents in both groups, the cross-sectional design, self-report measures, absence of objective smartphone use data, non-validated measures, possible selection and response bias, confounding, multiple statistical testing, differences in questionnaire administration between age groups, and the fact that data were collected during March–May 2021, when the COVID-19 pandemic may have substantially affected smartphone use, schooling, sleep, and mental health. The use of non-probability convenience sampling may have introduced selection and self-selection bias, and the resulting sample should not be considered nationally representative, despite responses being obtained from participants across Romania. Problematic smartphone use or smartphone addiction was not assessed using a specific validated diagnostic instrument. Therefore, the duration of daily smartphone use reported in the present study should not be interpreted as an indicator of smartphone addiction. Future studies should combine objective or self-reported measures of smartphone use duration with validated instruments assessing problematic smartphone use and associated functional impairment. At the same time, because the duration of use was assessed as overall daily smartphone use rather than separately for each type of activity, our data do not allow us to determine the amount of time spent on individual smartphone activities or to examine their independent associations with the investigated outcomes. Sleep characteristics and mental-health-related experiences were assessed using individual self-reported questionnaire items rather than validated multidimensional instruments. Consequently, although the questionnaire allowed the evaluation of several distinct aspects of sleep and emotional well-being, it did not permit the calculation of validated sleep quality, daytime sleepiness, depression, or anxiety scores, nor did it allow clinical diagnoses to be established. The findings should therefore be interpreted as associations with individual self-reported sleep and emotional indicators. Furthermore, due to the cross-sectional design of the study, temporal or causal relationships between smartphone use and the investigated sleep, physical, and emotional outcomes cannot be established. Therefore, the observed findings should be interpreted as associations rather than evidence of causal effects. An additional limitation concerns differences in the source of information and questionnaire administration between the two age groups. Children aged 7–12 years completed the questionnaire with parental assistance, with some items being explicitly parent-reported, whereas adolescents aged 13–18 years completed the questionnaire independently. This may have introduced informant and reporting bias, particularly for subjective sleep-related and emotional or anxiety-related experiences. Therefore, between-group differences should be interpreted cautiously, as they may reflect not only developmental differences but also differences in informants and response procedures. The design is cross-sectional and the analyses were exploratory, so causality cannot be inferred and the somatic findings, which did not survive correction for multiple testing, should be regarded as hypothesis-generating.

Nevertheless, to the best of our knowledge, this is among the few studies on Romanian children that underlines the effects of phone use on physical and mental health. Furthermore, it is most-likely the first study to assess the impact of excessive screen time in Romanian younger school-aged children, between 7 and 12 years of age.

## 5. Conclusions

Both children and adolescents are aware of and able to recognize the adverse effects of smartphone use; nevertheless, they spend several hours engaged in various smartphone-related activities and report insufficient sleep, restless and interrupted sleep, and disturbing dreams. They frequently wake up feeling tired and experience daytime sleepiness, despite continuing to use their smartphones before bedtime. Somatic complaints are common, particularly fatigue and exhaustion, as well as ocular discomfort, including eye pain and a burning sensation. Adolescent girls spend considerably more time using their smartphones than boys and report psychological and somatic symptoms more frequently. A substantial number of adolescents report some degree of emotional- and anxiety-related symptoms/experiences that are suggestive for depressive and anxiety symptoms, which appear to be associated with both smartphone use before bedtime and the amount of time spent using smartphones. Our findings highlight the need to develop appropriate preventive strategies to mitigate the adverse effects of smartphone use.

## Figures and Tables

**Table 1:** Sociodemographic characteristics of participants in the 7–12-year-old group (n = 171).

Variables	Number (%)
**Gender**	
Male	85 (49.71%)
Female	86 (50.29%)
**Body mass index (BMI)**	
Normal	108 (63.16%)
Obesity	21 (12.28%)
Underweight	13 (7.60%)
Overweight	29 (16.96%)
**Grade**	
First	15 (8.77%)
Second	27 (15.79%)
Third	24 (14.04%)
Fourth	24 (14.04%)
Fifth	26 (15.20%)
Sixth	41 (23.98%)
Seventh	14 (8.19%)
**Educational unit**	
Primary school	91 (53.22%)
Gymnasium	80 (46.78%)
**Place of residence**	
Rural	73 (42.69%)
Urban	98 (57.31%)
**Mother’s highest educational level**	
Without studies	2 (1.17%)
University degree	73 (42.69%)
Gymnasium	58 (33.92%)
Primary school	13 (7.60%)
Post-secondary studies	25 (14.62%)
**Father’s highest educational level**	
University degree	61 (35.67%)
Gymnasium	90 (52.63%)
Primary school	10 (5.85%)
Post-secondary studies	10 (5.85%)

Notes: BMI, body mass index.

**Table 2:** Smartphone use, sleep characteristics, and reported effects in the 7–12-year-old group (n = 171).

Variables	Number (%)
**What kind of phone do you use?**	
Smartphone	171 (100.00%)
Classic phone	0 (0.00%)
I do not have a phone	0 (0.00%)
**How long have you had a phone?**	
<1 year	56 (32.75%)
1–3 years	80 (46.78%)
3–5 years	29 (16.96%)
>5 years	6 (3.51%)
**Why did you get the phone?**	
Keeping in touch with family	116 (67.84%)
Keeping in touch with friends	49 (28.65%)
To learn	69 (40.35%)
Play, have fun	63 (36.84%)
Other	2 (1.17%)
**When do you use your phone most?**	
At the weekend	61 (35.67%)
During the week	110 (64.33%)
**How many hours a day do you use your phone?**	
<1 h	15 (8.77%)
1–3 h	69 (40.35%)
3–5 h	58 (33.92%)
>5 h	29 (16.96%)
**What do you use the phone for?**	
Calls	90 (52.63%)
SMS, online chat	90 (52.63%)
Social media	51 (29.82%)
Games	105 (61.40%)
Movies	37 (21.64%)
Listening to music	81 (47.37%)
Learning	140 (81.87%)
Other	4 (2.34%)
**Why is your phone important to you?**	
Obtaining information	106 (61.99%)
Communication	121 (70.76%)
Entertainment	88 (51.46%)
Other	7 (4.09%)
**Do you think you use your phone too much?**	
No	44 (25.70%)
Yes	127 (74.20%)
**Do you think your phone use affects you?**	
No	22 (12.80%)
Yes	149 (87.13%)
**What are the negative impacts of smartphone use?**	
Addiction	145 (84.80%)
Visual impairments	123 (71.93%)
Fake news	46 (26.90%)
Bullying	60 (35.09%)
Sleeping disorders	50 (29.24%)
Disconnection from reality	99 (57.89%)
Other	3 (1.75%)
**Have you noticed the negative effects of phone use on other children?**	
No	45 (26.32%)
Yes	126 (73.68%)
**Do you talk to your child about the correct use of the phone? (Question to parents—QP)**	
No	18 (10.53%)
Yes	153 (89.47%)
**Has your child received any useful advice at school about using the phone properly? (QP)**	
No	104 (60.82%)
Yes	67 (39.18%)
**Is it important that your phone is expensive and the latest?**	
No	123 (71.93%)
Yes	48 (28.07%)
**Would you take your child’s phone to punish him/her? (QP)**	
No	57 (33.33%)
Yes	114 (66.67%)
**When are you going to bed?**	
00:00	2 (1.17%)
00:30	1 (0.58%)
01:30	2 (1.17%)
20:00	1 (0.58%)
20:30	1 (0.58%)
21:00	28 (16.37%)
21:30	13 (7.60%)
22:00	70 (40.94%)
22:30	16 (9.36%)
23:00	31 (18.13%)
23:30	6 (3.51%)
**Is the child getting enough sleep according to NSF recommendations? (QP)**	
No	81 (47.37%)
Yes	90 (52.63%)
**What do you do before you go to sleep?**	
Use my phone	63 (36.84%)
Read	49 (28.65%)
Watch TV	106 (61.99%)
Other	25 (14.62%)
**Do you wake up during the night?**	
No	124 (72.51%)
Yes	47 (27.49%)
**How often do you wake up during the night?**	
Repeatedly	7 (14.89%)
Once	40 (85.22%)
**Do you have nightmares?**	
No	107 (62.57%)
Yes	64 (37.43%)
**When you wake up, do you feel that you have not rested?**	
No	77 (45.03%)
Yes	94 (54.97%)
**Are you sleepy or do you sleep during the day?**	
I am not sleepy	91 (53.22%)
Yes, I am sleepy, but I do not sleep during the day	58 (33.92%)
Yes, I am sleepy, and I sleep during the day	22 (12.87%)
**During or after using the phone, you:**	
Feel tired, exhausted	79 (46.20%)
Feel dizzy	10 (5.85%)
Have a headache	54 (31.58%)
Have a burning sensation/pain in your eyes	27 (15.79%)
Have blurry vision	18 (10.53%)
Feel pain in your wrist	14 (8.19%)
Feel pain in your thumb	6 (3.51%)
None	53 (30.99%)
**Do you ever get the feeling that your phone is vibrating in your pocket?**	
No	152 (88.89%)
Yes	19 (11.11%)
**Do you ever get the feeling that you hear your phone ringing?**	
No	142 (83.04%)
Yes	29 (16.96%)
**Choose which of the following has happened to your child (QP)**	
Feeling sad	36 (21.05%)
Did not feel like playing or having fun	38 (22.22%)
Felt like they were doing everything wrong	37 (21.64%)
Cried for no reason	34 (19.88%)
Felt lonely	26 (15.20%)
Felt they would never be as good as others	46 (26.90%)
None	73 (42.69%)
**Is your child anxious? (QP)**	
No	77 (45.03%)
Yes	94 (54.97%)
**When scared, the child:**	
Sweats a lot	29 (16.96%)
Breathes harder	14 (8.19%)
Heart beats faster	100 (58.48%)
None	54 (31.58%)
**Are you afraid to be alone at home?**	
No	131 (76.61%)
Yes	40 (23.39%)
**Are you scared if you do not sleep at home?**	
No	150 (87.72%)
Yes	21 (12.28%)
**Do you find it difficult to talk to strangers?**	
No	110 (64.33%)
Yes	61 (35.67%)
**Are you anxious about performing in front of others?**	
No	73 (42.69%)
Yes	98 (57.31%)
**Do you worry about whether others like you?**	
No	92 (53.80%)
Yes	79 (46.20%)

Notes: NSF, National Sleep Foundation. Percentages for questions allowing multiple responses may sum to more than 100%.

**Table 3:** Sociodemographic characteristics of participants in the 13–18-year-old group (n = 213).

Variables	Number (%)
**Gender**	
Male	68 (31.92%)
Female	145 (68.08%)
**BMI**	
Normal	179 (84.04%)
Obesity	7 (3.29%)
Underweight	5 (2.35%)
Overweight	22 (10.33%)
**Grade**	
Sixth	3 (1.41%)
Seventh	41 (19.25%)
Eighth	15 (7.04%)
Ninth	32 (15.02%)
Tenth	50 (23.47%)
Eleventh	37 (17.37%)
Twelfth	35 (16.43%)
**Educational unit**	
Gymnasium	59 (27.70%)
High school	143 (67.14%)
Vocational school	11 (5.16%)
**Place of residence**	
Rural	94 (44.13%)
Urban	119 (55.87%)
**Mother’s highest educational level**	
University degree	75 (35.21%)
Gymnasium	10 (4.69%)
High school	96 (45.07%)
Post-secondary studies	32 (15.02%)
**Father’s highest educational level**	
University degree	60 (28.17%)
Gymnasium	12 (5.63%)
High school	120 (56.34%)
Post-secondary studies	21 (9.86%)

Notes: BMI, body mass index.

**Table 4:** Smartphone use, sleep characteristics, and reported effects in the 13–18-year-old group (n = 213).

Variables	Number (%)
**What kind of phone do you use?**	
Smartphone	213 (100.00%)
Classic phone	0 (0.00%)
I do not have a phone	0 (0.00%)
**How long have you been using a smartphone?**	
<1 year	5 (2.35%)
1–3 years	26 (12.21%)
3–5 years	83 (38.97%)
>5 years	99 (46.48%)
**Why did you get a phone?**	
Keeping in touch with family	193 (90.61%)
Keeping in touch with friends	128 (60.09%)
To learn	76 (35.68%)
Play/have fun	76 (35.68%)
Other	1 (0.47%)
**When do you use your phone most?**	
Weekend	67 (31.46%)
During week	146 (68.54%)
**How many hours per day do you use your smartphone?**	
<1 h	7 (3.29%)
1–3 h	62 (29.11%)
3–5 h	87 (40.85%)
>5 h	57 (26.76%)
**What do you use your smartphone for?**	
Calls	176 (82.63%)
SMS/online chat	198 (92.96%)
Social media	190 (89.20%)
Games	91 (42.72%)
Movies	59 (27.70%)
Listening to music	172 (80.75%)
Learning	182 (85.45%)
Other	3 (1.41%)
**Why is your phone important to you?**	
Obtaining information	173 (81.22%)
Communication	206 (96.71%)
Entertainment	138 (64.79%)
Other	0 (0.00%)
**Do you think you use your smartphone too much?**	
No	58 (27.23%)
Yes	155 (72.77%)
**Do you think phone use affects you?**	
No	43 (20.19%)
Yes	170 (79.81%)
**What negative impacts can smartphone use have?**	
Addiction	190 (89.20%)
Visual impairment	149 (69.95%)
Fake news	76 (35.68%)
Bullying	104 (48.83%)
Sleeping disorders	88 (41.31%)
Disconnection from reality	116 (54.46%)
Other	2 (0.94%)
**Have you noticed negative effects of smartphone use on other children?**	
No	78 (36.62%)
Yes	135 (63.38%)
**Have you talked with your parents about correct smartphone use?**	
No	105 (49.30%)
Yes	108 (50.70%)
**Have you received useful advice at school about smartphone use?**	
No	124 (58.22%)
Yes	89 (41.78%)
**Is it important for your phone to be expensive/the latest model?**	
No	157 (73.71%)
Yes	56 (26.29%)
**Do your parents take away your phone as punishment?**	
No	160 (75.12%)
Yes	53 (24.88%)
**What time do you usually go to bed?**	
00:00	31 (14.55%)
00:30	5 (2.35%)
01:00	9 (4.23%)
01:30	3 (1.41%)
02:00	4 (1.88%)
02:30	2 (0.94%)
03:30	1 (0.47%)
21:00	5 (2.35%)
21:30	6 (2.82%)
22:00	30 (14.08%)
22:30	22 (10.33%)
23:00	66 (30.99)
23:30	29 (13.62%)
**Do you sleep enough according to NSF recommendations?**	
No	115 (53.99%)
Yes	98 (46.01%)
**What do you usually do before sleep?**	
Use phone	162 (76.06%)
Read	69 (32.39%)
Watch TV	90 (42.25%)
Other	20 (9.39%)
**Do you wake during the night?**	
No	118 (55.40%)
Yes	95 (44.60%)
**How often do you wake up during the night?**	
Repeatedly	29 (30.53%)
Once	66 (69.47%)
**Do you have nightmares?**	
No	101 (47.42%)
Yes	112 (52.58%)
**Do you wake feeling unrefreshed?**	
No	38 (17.84%)
Yes	175 (82.16%)
**Daytime sleepiness**	
Sleepy but no daytime sleep	119 (55.87%)
Sleepy and sleeps during day	50 (23.47%)
Not sleepy	44 (20.66%)
**Symptoms during/after phone use**	
Tired/exhausted	85 (39.91%)
Dizzy	25 (11.74%)
Headache	66 (30.99%)
Burning/pain eyes	72 (33.80%)
Blurry vision	45 (21.13%)
Wrist pain	20 (9.39%)
Thumb pain	28 (13.15%)
None	50 (23.47%)
**Phantom vibration**	
No	130 (61.03%)
Yes	83 (38.97%)
**Phantom ringing**	
No	140 (65.73%)
Yes	73 (34.27%)
**Feelings**	
Sadness	95 (44.60%)
Do not feel like playing/having fun	48 (22.54%)
Do everything wrong	83 (38.97%)
Cried for no reason	49 (23.00%)
Loneliness	85 (39.91%)
Never as good as others	92 (43.19%)
None	58 (27.23%)
**Do you consider yourself an anxious person?**	
No	62 (29.11%)
Yes	151 (70.89%)
**If you are scared, what do you experience?**	
Sweating	44 (20.66%)
Breathing harder	46 (21.60%)
Heart beating faster	178 (83.57%)
None	24 (11.27%)
**Are you afraid when you are alone at home?**	
No	183 (85.92%)
Yes	30 (14.08%)
**Are you scared if you do not sleep at home?**	
No	205 (96.24%)
Yes	8 (3.76%)
**Is it difficult for you to talk to strangers?**	
No	138 (64.79%)
Yes	75 (35.21%)
**Are you anxious about performing in front of others?**	
No	69 (32.39%)
Yes	144 (67.61%)
**Do you worry whether others like you?**	
No	87 (40.85%)
Yes	126 (59.15%)

Notes: NSF, National Sleep Foundation. Percentages for questions allowing multiple responses may sum to more than 100%.

**Table 5:** Correlation between years of smartphone ownership and daily smartphone use.

	How Many Hours They Use the Smartphone Daily?			
How Long Have They Had the Smartphone?	<1 h/Day	1–3 h/Day	3–5 h/Day	>5 h/Day
Group 1				
<1 year	9 (60.00%)	28 (40.58%)	15 (25.86%)	4 (13.79%)
1–3 years	4 (26.67%)	33 (47.83%)	30 (51.72%)	13 (44.83%)
3–5 years	1 (6.67%)	8 (11.59%)	10 (17.24%)	10 (34.48%)
>5 years	1 (6.67%)	0 (0.00%)	3 (5.17%)	2 (6.90%)
Total	15 (100.00%)	69 (100.00%)	58 (100.00%)	29 (100.00%)
Group 2				
<1 year	1 (14.29%)	3 (4.84%)	0 (0.00%)	1 (1.75%)
1–3 years	3 (42.86%)	12 (19.35%)	8 (9.20%)	3 (5.26%)
3–5 years	2 (28.57%)	26 (41.94%)	29 (33.33%)	26 (45.61%)
>5 years	1 (14.29%)	21 (33.87%)	50 (57.47%)	27 (47.37%)
Total	7 (100.00%)	62 (100.00%)	87 (100.00%)	57 (100.00%)

**Table 6:** Age and daily smartphone use—group 1.

Group 1				
	**How Many Hours They Use the Smartphone Daily?**			
**Age 7–12 Years**	**<1 h/Day**	**1–3 h/Day**	**3–5 h/Day**	**>5 h/Day**
Number of values	15	69	58	29
Minimum	7.000	7.000	7.000	9.000
Median	10.00	10.00	11.00	12.00
Maximum	12.00	12.00	12.00	12.00
Mean	9.667	10.01	10.31	11.52
Std. Deviation	1.799	1.736	1.581	0.7847
**Comparison**				***p*-Value**
<1 h/day vs. 1–3 h/day				0.4953
<1 h/day vs. 3–5 h/day				0.2183
<1 h/day vs. >5 h/day				**0.0003**
1–3 h/day vs. 3–5 h/day				0.3922
1–3 h/day vs. >5 h/day				**<0.0001**
3–5 h/day vs. >5 h/day				**0.0005**

Statistically significant *p* < 0.05. Values in bold indicate statistically significant results.

**Table 7:** Age and daily smartphone use—group 2.

Group 2				
	**How Many Hours They Use the Smartphone Daily?**			
**Age 13–18 Years**	**<1 h/Day**	**1–3 h/Day**	**3–5 h/Day**	**>5 h/Day**
Number of values	7	62	87	57
Minimum	13.00	13.00	13.00	13.00
Median	14.00	15.50	16.00	16.00
Maximum	18.00	18.00	18.00	18.00
Mean	15.00	15.56	16.00	15.58
Std. Deviation	1.732	1.675	1.494	1.592
**Comparison**				***p*-Value **
<1 h/day vs. 1–3 h/day				0.3898
<1 h/day vs. 3–5 h/day				0.1173
<1 h/day vs. >5 h/day				0.3670
1–3 h/day vs. 3–5 h/day				0.0990
1–3 h/day vs. >5 h/day				0.9269
3–5 h/day vs. >5 h/day				0.1408

Statistically significant *p* < 0.05.

**Table 8:** Sleep duration and years of smartphone ownership.

Dunn’s Multiple Comparison Test		
**Comparison**	**Group 1** ***p*-Value**	**Group 2** ***p*-Value**
<1 year vs. 1–3 years	**0.0029**	0.7445
<1 year vs. 3–5 years	**0.0211**	0.0777
<1 year vs. >5 years	**0.0024**	**0.0215**
1–3 years vs. 3–5 years	0.6599	**0.0059**
1–3 years vs. >5 years	**0.0183**	**<0.0001**
3–5 years vs. >5 years	0.1095	0.0522

Notes: Values in bold indicate statistically significant results.

**Table 9:** Sleep duration and hours of smartphone use per day.

Dunn’s Multiple Comparison Test		
**Comparison**	**Group 1** ***p*-Value**	**Group 2** ***p*-Value**
<1 h/day vs. 1–3 h/day	0.4374	0.3135
<1 h/day vs. 3–5 h/day	0.5562	**0.0486**
<1 h/day vs. >5 h/day	0.8298	**0.0434**
1–3 h/day vs. 3–5 h/day	0.1078	**0.0079**
1–3 h/day vs. >5 h/day	0.2669	**0.0048**
3–5 h/day vs. >5 h/day	0.8803	0.6009

Notes: Values in bold indicate statistically significant results.

**Table 10:** BMI and daily phone use.

Group 1				
	**How Many Hours They Use the Phone?**			
**BMI**	**<1 h/Day**	**1–3 h/Day**	**3–5 h/Day**	**>5 h/Day**
Number of values	15	69	58	29
Minimum	13.19	12.62	13.39	15.56
Median	16.46	17.75	19.35	20.71
Maximum	18.93	38.10	29.08	31.22
Mean	16.30	18.09	19.57	20.94
Std. Deviation	1.822	3.835	3.686	3.464

**Table 11:** Dunn’s Multiple Comparison test BMI and daily smartphone use.

Dunn’s Multiple Comparison Test	
**Comparison**	***p*-Value**
<1 h/day vs. 1–3 h/day	0.0569
<1 h/day vs. 3–5 h/day	**0.0009**
<1 h/day vs. >5 h/day	**<0.0001**
1–3 h/day vs. 3–5 h/day	**0.0207**
1–3 h/day vs. >5 h/day	**<0.0001**
3–5 h/day vs. >5 h/day	**0.0471**

Notes: Values in bold indicate statistically significant results.

**Table 12:** Multivariable logistic regression models: crude and adjusted odds ratios for the associations reported in the manuscript.

Group	Outcome	Exposure	Events/n	Crude OR (95% CI)	Adjusted OR (95% CI)	*p*	q (BH)	Model	EPV
**Sleep outcomes**									
**Group 1 (7–12 y)**	Daytime sleepiness	Daily smartphone use > 3 h	80/171	1.82 (0.99–3.35)	1.67 (0.86–3.25)	0.131	0.181	full	13.3
	Daytime sleepiness	Smartphone use before bedtime	80/171	**2.39 (1.27–4.55)**	**2.06 (1.03–4.15)**	**0.041**	0.082	full	13.3
	Disturbing dreams	Daily smartphone use > 3 h	64/171	1.15 (0.62–2.15)	1.26 (0.64–2.49)	0.508	0.508	full	10.7
	Insufficient sleep (below NSF recommendation)	Daily smartphone use > 3 h	81/171	**2.10 (1.14–3.89)**	1.54 (0.77–3.09)	0.220	0.283	full	16.2
	Insufficient sleep (below NSF recommendation)	Smartphone use before bedtime	81/171	**2.84 (1.51–5.47)**	**2.58 (1.27–5.35)**	**0.009**	**0.041**	full	16.2
	Nocturnal awakenings	Daily smartphone use > 3 h	47/171	0.71 (0.36–1.39)	0.68 (0.32–1.42)	0.311	0.349	red.	11.8
	Nocturnal awakenings	Smartphone use before bedtime	47/171	1.58 (0.79–3.13)	1.86 (0.88–3.95)	0.103	0.154	red.	11.8
	Non-restorative sleep	Daily smartphone use > 3 h	94/171	**2.41 (1.31–4.51)**	1.91 (0.98–3.75)	0.058	0.105	full	12.8
	Non-restorative sleep	Smartphone use before bedtime	94/171	**3.38 (1.75–6.77)**	**2.92 (1.43–6.15)**	**0.004**	**0.041**	full	12.8
**Group 2 (13–18 y)**	Daytime sleepiness	Daily smartphone use > 3 h	169/213	**3.74 (1.89–7.53)**	**2.35 (1.10–5.04)**	**0.027**	0.070	red.	11
	Daytime sleepiness	Smartphone use before bedtime	169/213	**3.25 (1.59–6.63)**	**2.28 (1.03–4.99)**	**0.040**	0.082	red.	11
	Disturbing dreams	Daily smartphone use > 3 h	112/213	**3.24 (1.79–5.99)**	**2.43 (1.26–4.75)**	**0.008**	**0.041**	full	16.8
	Insufficient sleep (below NSF recommendation)	Daily smartphone use > 3 h	115/213	**2.68 (1.49–4.88)**	**2.50 (1.32–4.83)**	**0.006**	**0.041**	full	19.6
	Insufficient sleep (below NSF recommendation)	Smartphone use before bedtime	115/213	1.60 (0.85–3.03)	1.49 (0.75–3.00)	0.258	0.310	full	19.6
	Nocturnal awakenings	Daily smartphone use > 3 h	95/213	**2.42 (1.33–4.50)**	**2.18 (1.13–4.29)**	**0.021**	0.070	full	15.8
	Nocturnal awakenings	Smartphone use before bedtime	95/213	1.08 (0.57–2.06)	0.79 (0.39–1.58)	0.506	0.508	full	15.8
	Non-restorative sleep	Daily smartphone use > 3 h	175/213	**3.27 (1.60–6.80)**	2.05 (0.93–4.53)	0.074	0.121	red.	9.5
	Non-restorative sleep	Smartphone use before bedtime	175/213	**2.91 (1.37–6.11)**	**2.58 (1.13–5.87)**	**0.024**	0.070	red.	9.5
**Somatic outcomes**									
**Group 1 (7–12 y)**	Any somatic complaint	Daily smartphone use > 3 h	118/171	1.73 (0.90–3.36)	1.88 (0.94–3.84)	0.079	0.260	red.	13.2
	Any somatic complaint	Smartphone use before bedtime	118/171	1.35 (0.69–2.73)	1.22 (0.59–2.56)	0.597	0.661	red.	13.2
	Fatigue/tiredness	Daily smartphone use > 3 h	79/171	**2.10 (1.15–3.91)**	**2.46 (1.27–4.89)**	**0.009**	0.102	full	13.2
	Fatigue/tiredness	Smartphone use before bedtime	79/171	1.34 (0.72–2.51)	1.26 (0.63–2.52)	0.522	0.661	full	13.2
	Headache	Daily smartphone use > 3 h	54/171	1.83 (0.96–3.57)	1.71 (0.86–3.46)	0.131	0.260	red.	13.5
	Ocular symptoms	Daily smartphone use > 3 h	37/171	1.02 (0.49–2.13)	1.05 (0.48–2.30)	0.896	0.896	red.	9.2
**Group 2 (13–18 y)**	Any somatic complaint	Daily smartphone use > 3 h	163/213	**2.17 (1.12–4.17)**	1.46 (0.71–2.98)	0.296	0.444	red.	12.5
	Any somatic complaint	Smartphone use before bedtime	163/213	1.95 (0.96–3.90)	1.74 (0.81–3.67)	0.152	0.260	red.	12.5
	Fatigue/tiredness	Daily smartphone use > 3 h	85/213	**2.47 (1.33–4.72)**	1.69 (0.85–3.42)	0.138	0.260	full	14.2
	Fatigue/tiredness	Smartphone use before bedtime	85/213	**2.06 (1.05–4.22)**	1.92 (0.92–4.17)	0.087	0.260	full	14.2
	Headache	Daily smartphone use > 3 h	66/213	**2.51 (1.29–5.18)**	2.09 (1.00–4.54)	0.054	0.260	full	11
	Ocular symptoms	Daily smartphone use > 3 h	95/213	1.52 (0.85–2.76)	1.19 (0.62–2.28)	0.606	0.661	full	15.8
**Psychological outcomes**									
**Group 1 (7–12 y)**	Any depressive symptom	Daily smartphone use > 3 h	98/171	1.64 (0.89–3.03)	1.39 (0.72–2.70)	0.330	0.496	full	12.2
	Any depressive symptom	Smartphone use before bedtime	98/171	**2.59 (1.35–5.13)**	**2.38 (1.18–4.94)**	**0.018**	0.070	full	12.2
	Phantom ringing	Daily smartphone use > 3 h	29/171	**2.49 (1.09–6.09)**	1.82 (0.75–4.63)	0.194	0.333	red.	7.2
	Phantom vibration	Daily smartphone use > 3 h	19/171	1.76 (0.67–4.96)	1.07 (0.37–3.20)	0.900	0.927	red.	4.8
	Phantom vibration	Smartphone use before bedtime	19/171	**4.42 (1.64–13.22)**	**3.82 (1.36–11.93)**	**0.014**	0.070	red.	4.8
	Self-reported anxiety	Daily smartphone use > 3 h	94/171	1.23 (0.67–2.25)	0.93 (0.48–1.81)	0.837	0.927	full	12.8
**Group 2 (13–18 y)**	Any depressive symptom	Daily smartphone use > 3 h	155/213	**3.19 (1.70–6.02)**	1.84 (0.89–3.76)	0.097	0.193	red.	14.5
	Any depressive symptom	Smartphone use before bedtime	155/213	**2.36 (1.21–4.61)**	**2.23 (1.01–4.92)**	**0.045**	0.136	red.	14.5
	Phantom ringing	Daily smartphone use > 3 h	73/213	**2.17 (1.15–4.26)**	1.97 (0.98–4.09)	0.063	0.151	full	12.2
	Phantom vibration	Daily smartphone use > 3 h	83/213	1.57 (0.86–2.91)	1.03 (0.53–2.04)	0.927	0.927	full	13.8
	Phantom vibration	Smartphone use before bedtime	83/213	**3.36 (1.63–7.52)**	**2.91 (1.36–6.70)**	**0.008**	0.070	full	13.8
	Self-reported anxiety	Daily smartphone use > 3 h	151/213	1.81 (0.98–3.36)	1.15 (0.57–2.30)	0.692	0.922	full	10.3
**Use pattern outcomes**									
**Group 1 (7–12 y)**	Smartphone use for gaming	Male sex	105/171	**3.34 (1.77–6.50)**	**3.55 (1.83–7.09)**	**<0.001**	**<0.001**	full	16.5
**Group 2 (13–18 y)**	Smartphone use for gaming	Male sex	91/213	1.69 (0.94–3.03)	**2.02 (1.08–3.82)**	**0.028**	**0.028**	full	22.8

Note. Bold numbers indicate nominal statistical significance (*p* < 0.05), based on the corresponding 95% confidence interval or *p* value; bold q values indicate significance after Benjamini–Hochberg correction (q < 0.05). OR, odds ratio; CI, confidence interval; BH, Benjamini–Hochberg; EPV, events per variable; NSF, National Sleep Foundation; red., reduced model. Multivariable logistic regression models: crude and adjusted odds ratios for the associations reported in the manuscript. Adjusted models include age (continuous), sex, place of residence, sleep duration (continuous, omitted where the outcome derives from it), and mutual adjustment for the alternative exposure. “Reduced” models include age, sex, and the alternative exposure only, which is applied automatically where the full model would yield fewer than ten events per variable (EPV). Confidence intervals are profile-likelihood-based. q = *p*-value adjusted within the domain using the Benjamini–Hochberg procedure.

**Table 13:** Prevalence of each outcome across the four categories of daily smartphone use, with tests for trend and for departure from linearity.

Group	Outcome	Daily Smartphone Use Duration				OR per Category (95% CI)	*p* Trend	*p* Non-Linearity
		<1 h/day	1–3 h/day	3–5 h/day	>5 h/day			
**Group 1 (7–12 y)**	Insufficient sleep (below NSF recommendation)	5/15 (33.3)	27/69 (39.1)	34/58 (58.6)	15/29 (51.7)	1.01 (0.68–1.51)	0.949	0.110
	Nocturnal awakenings	7/15 (46.7)	19/69 (27.5)	15/58 (25.9)	6/29 (20.7)	0.70 (0.45–1.09)	0.122	0.593
	Non-restorative sleep	4/15 (26.7)	33/69 (47.8)	38/58 (65.5)	19/29 (65.5)	1.47 (0.99–2.22)	0.060	0.414
	Disturbing dreams	8/15 (53.3)	22/69 (31.9)	25/58 (43.1)	9/29 (31.0)	0.96 (0.64–1.42)	0.829	0.200
	Daytime sleepiness	4/15 (26.7)	29/69 (42.0)	31/58 (53.4)	16/29 (55.2)	1.36 (0.92–2.04)	0.122	0.743
	Any somatic complaint	8/15 (53.3)	45/69 (65.2)	42/58 (72.4)	23/29 (79.3)	**1.60 (1.06–2.46)**	**0.029**	0.967
	Fatigue/tiredness	5/15 (33.3)	26/69 (37.7)	30/58 (51.7)	18/29 (62.1)	**1.79 (1.20–2.70)**	**0.005**	0.855
	Any depressive symptom	7/15 (46.7)	36/69 (52.2)	36/58 (62.1)	19/29 (65.5)	1.22 (0.83–1.81)	0.316	0.972
	Self-reported anxiety	5/15 (33.3)	39/69 (56.5)	30/58 (51.7)	20/29 (69.0)	1.16 (0.78–1.72)	0.465	0.293
**Group 2 (13–18 y)**	Insufficient sleep (below NSF recommendation)	3/7 (42.9)	23/62 (37.1)	51/87 (58.6)	38/57 (66.7)	**1.60 (1.11–2.34)**	**0.013**	0.374
	Nocturnal awakenings	2/7 (28.6)	19/62 (30.6)	45/87 (51.7)	29/57 (50.9)	**1.45 (1.01–2.09)**	**0.046**	0.331
	Non-restorative sleep	2/7 (28.6)	46/62 (74.2)	78/87 (89.7)	49/57 (86.0)	1.47 (0.91–2.41)	0.120	0.083
	Disturbing dreams	0/7 (0.0)	23/62 (37.1)	49/87 (56.3)	40/57 (70.2)	**1.89 (1.30–2.81)**	**0.001**	0.242
	Daytime sleepiness	3/7 (42.9)	41/62 (66.1)	75/87 (86.2)	50/57 (87.7)	**1.63 (1.02–2.63)**	**0.043**	0.674
	Any somatic complaint	4/7 (57.1)	42/62 (67.7)	70/87 (80.5)	47/57 (82.5)	1.22 (0.80–1.87)	0.362	0.884
	Fatigue/tiredness	1/7 (14.3)	17/62 (27.4)	37/87 (42.5)	30/57 (52.6)	1.43 (0.98–2.10)	0.069	0.977
	Any depressive symptom	3/7 (42.9)	36/62 (58.1)	69/87 (79.3)	47/57 (82.5)	1.36 (0.89–2.12)	0.161	0.696
	Self-reported anxiety	4/7 (57.1)	39/62 (62.9)	69/87 (79.3)	39/57 (68.4)	0.88 (0.59–1.32)	0.549	0.208

Note: Bold numbers indicate nominally statistically significant results (*p* < 0.05).

## Data Availability

The data presented in this article are available upon request from the corresponding author. The data are not publicly available due to patient privacy and ethical restrictions.
